# Optimizing Brain Biology Through Near-Infrared-Induced Mitochondrial Melatonin Synthesis: A Hypothesis Paper

**DOI:** 10.7759/cureus.105322

**Published:** 2026-03-16

**Authors:** Joseph Mercola

**Affiliations:** 1 Family Medicine, Midwestern University Chicago College of Osteopathic Medicine, Downers Grove, USA

**Keywords:** alzheimer's disease, cytochrome c oxidase, glutathione, mitochondrial melatonin, near-infrared light, neuroprotection, photobiomodulation

## Abstract

The human brain consumes approximately 20% of total energy production despite comprising only 2% of body mass, rendering neurons particularly vulnerable to oxidative damage. Modern indoor lifestyles have dramatically reduced exposure to near-infrared (NIR) radiation, a component of sunlight that penetrates biological tissues. Concurrently, age-related declines in both pineal melatonin production and mitochondrial function have been implicated in the pathogenesis of neurodegenerative diseases. Additionally, aging is associated with declining availability of glutathione precursors, particularly glycine and cysteine, which may limit endogenous antioxidant responses even when enzymatic capacity is preserved.

This hypothesis paper synthesizes evidence from photobiomodulation (PBM) research, mitochondrial biology, and melatonin biochemistry to propose a mechanistic framework whereby NIR radiation activates mitochondrial melatonin synthesis, potentially triggering an antioxidant cascade that may confer neuroprotection. The framework explicitly incorporates the requirement for adequate glutathione precursor substrate availability as a potential rate-limiting factor.

A targeted narrative synthesis informed the development of the proposed mechanistic framework. Peer-reviewed publications were identified through searches of PubMed, Web of Science, and Google Scholar (1990-2025) using terms related to PBM, mitochondrial melatonin, glutathione metabolism, and neuroprotection. Studies were selected based on relevance to the proposed framework, with emphasis on mechanistic studies, randomized controlled trials, and systematic reviews. Priority was given to publications from 2020 to 2025, while seminal foundational studies were retained regardless of publication date. Evidence supporting each component of the proposed cascade was categorized by strength to maintain transparency regarding the distinction between established findings and untested hypotheses.

The proposed NIR-mitochondrial melatonin-glutathione cascade represents a biologically plausible mechanism for endogenous neuroprotection, contingent upon adequate substrate availability. While substantial evidence supports individual components, the integrated hypothesis requires rigorous experimental validation. Concurrent attention to glutathione precursor status through glycine and N-acetylcysteine (NAC) supplementation may be necessary to realize the full therapeutic potential of this approach.

## Introduction and background

Photobiomodulation (PBM) refers to the therapeutic application of red and near-infrared (NIR) light, typically in the wavelength range of 620-1100 nm, to biological tissues. Originally investigated for wound healing and pain management, PBM has increasingly been studied for neurological applications, including transcranial delivery targeting brain tissue. The biological basis for PBM is thought to involve the absorption of photons by mitochondrial chromophores, particularly cytochrome c oxidase (CCO), leading to enhanced mitochondrial function and downstream cellular signaling. While individual components of PBM photobiology are well-characterized, a unified mechanistic framework linking NIR exposure to comprehensive neuroprotection has not been established. This paper proposes such a framework, integrating recent discoveries in mitochondrial melatonin biology with established PBM photobiology, and subjects it to critical evaluation, distinguishing established findings from untested hypotheses throughout.

The human brain represents one of the most metabolically demanding organs in the body, accounting for approximately 20% of total oxygen consumption and energy expenditure despite constituting only 2% of body mass [1]. This exceptional energy requirement is concentrated within neurons, each containing thousands of mitochondria operating near maximum capacity to sustain the electrochemical gradients essential for synaptic transmission and cognitive function [2]. The brain's vulnerability extends beyond its metabolic intensity; neuronal membranes are enriched with polyunsaturated fatty acids (PUFAs), rendering them particularly susceptible to lipid peroxidation, while the brain paradoxically maintains a lower baseline antioxidant capacity compared to other tissues [3].

The consequences of this metabolic precarity manifest progressively across the lifespan. Reactive oxygen species (ROS) accumulate as unavoidable byproducts of oxidative phosphorylation, inflicting progressive damage to mitochondrial membranes, proteins, and DNA [4]. Unlike most organelles, mitochondria lack catalase and depend entirely on glutathione peroxidase and related enzymes for hydrogen peroxide detoxification [5]. By the end of each waking day, measurable changes in brain metabolite levels occur, necessitating clearance processes during sleep [6]. Over decades, this cumulative oxidative burden contributes to the mitochondrial dysfunction characteristic of aging and neurodegenerative disease.

Modern human environments have diverged dramatically from the conditions under which our physiology evolved. Outdoor solar exposure, which provided substantial NIR radiation, has been largely replaced by indoor environments where NIR exposure is negligible [7,8]. This environmental shift may have consequences beyond vitamin D synthesis; emerging evidence suggests that NIR radiation interacts directly with mitochondrial chromophores, potentially serving as a physiological stimulus for endogenous protective mechanisms [9,10]. However, whether reduced NIR exposure in modern populations contributes to increased neurodegeneration remains speculative and has not been directly tested epidemiologically. Simultaneously, pineal melatonin production, long recognized as the primary source of a potent antioxidant, indoleamine, declines progressively with age, with pineal calcification accelerating this process [11]. In Alzheimer's disease, melatonin reduction is profound and appears to precede clinical symptoms [12,13].

The convergence of these observations suggests a potential mechanistic framework with significant implications for neuroprotection. Recent discoveries have fundamentally altered the understanding of melatonin biology. Studies by Suofu and colleagues demonstrated that mitochondria possess the complete enzymatic machinery for melatonin synthesis, including arylalkylamine N-acetyltransferase (AANAT) and acetylserotonin methyltransferase (ASMT), and that mitochondrial melatonin concentrations may exceed plasma levels by up to 100-fold in some experimental preparations, though concentrations vary considerably across tissues and measurement techniques [14,15]. Importantly, this extrapineal synthesis does not exhibit circadian rhythmicity and appears regulated by mechanisms distinct from the classical noradrenergic pathway controlling pineal production [15].

This hypothesis paper aims to synthesize these converging lines of evidence into a unified mechanistic framework. I propose that NIR radiation, absorbed by CCO in mitochondria, may initiate a signaling cascade hypothesized to culminate in local melatonin synthesis. If this local melatonin synthesis occurs, it could subsequently amplify antioxidant defenses through glutathione upregulation and sirtuin 3 (SIRT3)-mediated activation of superoxide dismutase 2 (SOD2). The framework integrates the established photobiology of CCO, the recently characterized mitochondrial melatonin synthesis pathway, and the well-documented antioxidant properties of melatonin into a coherent model with implications for understanding both normal brain aging and neurodegenerative pathology.

## Review

Methods

Scope and Approach

This paper is a hypothesis paper that synthesizes evidence from PBM research, mitochondrial biology, melatonin biochemistry, and clinical neuroscience to propose a mechanistic framework whereby NIR may activate mitochondrial melatonin synthesis with potential neuroprotective consequences. A hypothesis paper format was selected to facilitate integration across these traditionally separate domains and to articulate a testable mechanistic framework.

As a narrative synthesis, this work provides an interpretive integration of independently established findings rather than exhaustive systematic coverage of any single field. The manuscript's primary contribution lies in the novel connections proposed between fields, not in the comprehensive cataloguing of evidence within them. No quantitative pooling, meta-analysis, or statistical synthesis was performed. All evidence synthesis is narrative and qualitative. Readers should note that while individual components of the proposed framework have substantial empirical support, the complete integrated cascade has not been experimentally validated as a unified system in humans. The goal is to articulate a testable hypothesis that may guide future experimental investigation.

Literature Identification

Relevant literature was identified through searches of PubMed, Web of Science, and Google Scholar databases for articles published between 1990 and 2025. Search terms included the following: "photobiomodulation", "near-infrared", "low-level light therapy", "transcranial photobiomodulation", "cytochrome c oxidase", "mitochondrial melatonin", "extrapineal melatonin", "melatonin synthesis", "glutathione", "glutathione peroxidase", "glycine", "N-acetylcysteine", "SIRT3", "FOXO3", "FOXO3a", "superoxide dismutase", "neuroprotection", "neurodegeneration", "Alzheimer's disease", "Parkinson's disease", "oxidative stress", and "mitochondrial dysfunction". Terms were used in various combinations with iterative refinement based on initial results. Reference lists of key articles were manually searched for additional relevant studies. While the search included neurodegenerative disease terms broadly, the primary application context is Alzheimer's disease, where the convergence of mitochondrial dysfunction, melatonin deficiency, and oxidative stress is best documented.

Study Selection

Studies were selected based on the author's judgment regarding (1) relevance to one or more components of the proposed mechanistic framework connecting NIR radiation, mitochondrial function, melatonin synthesis, and antioxidant defense; (2) methodological quality, with preference for mechanistic studies using validated techniques, randomized controlled trials, and systematic reviews with meta-analyses; and (3) recency, with priority given to publications from 2020 to 2025 to incorporate the most current evidence. Seminal foundational works establishing core concepts were retained regardless of publication date given their essential contribution to the theoretical framework. This selection process reflects interpretive judgment appropriate to a hypothesis paper and does not constitute systematic screening. No formal inclusion/exclusion criteria, duplicate screening procedures, or study-level risk-of-bias assessments were applied. Final selection yielded 104 articles that inform this synthesis.

Evidence Categorization

To facilitate transparent evaluation of the proposed framework, evidence supporting each component was categorized by strength using a four-tier hierarchy designed for this manuscript. This hierarchy is not based on established clinical evidence grading frameworks such as the Grading of Recommendations Assessment, Development, and Evaluation (GRADE), which are designed for evaluating intervention efficacy from clinical trial evidence. Instead, it is designed to distinguish between mechanistic steps in a proposed cascade that have varying levels of experimental support:

★★★★ (Well-established): Multiple independent replications across laboratories and experimental systems; findings are broadly accepted in the relevant field.

★★★☆ (Supported, replication needed): Consistent findings from a limited number of studies or laboratories; the result is plausible and unrebutted but awaits broader replication.

★★☆☆ (Plausible hypothesis): Each individual mechanistic step in the proposed connection has independent empirical support, but the specific connection as formulated has not been directly tested experimentally.

★☆☆☆ (Speculative prediction): The proposed connection requires multiple untested inferential steps and lacks direct empirical support even for individual links in the chain; it represents a logical extrapolation from available data.

This hierarchy is the author's assessment, and readers should evaluate the cited evidence independently. Assignment to categories reflects judgment that may differ from other investigators' interpretations.

The brain's exceptional metabolic vulnerability

The human brain's exceptional metabolic demands, combined with its limited capacity for energy storage, create unique challenges for cellular homeostasis not shared by other tissues. Understanding this vulnerability provides essential context for appreciating why NIR-mediated neuroprotection mechanisms might have evolved and why their restoration could prove therapeutically valuable.

Neurons maintain extraordinarily high mitochondrial densities to support the continuous adenosine triphosphate (ATP) demands of synaptic transmission, ion gradient maintenance, and axonal transport [1,2]. A single cortical neuron may contain several thousand mitochondria, collectively accounting for a substantial fraction of neuronal cytoplasmic volume in metabolically active regions [16]. These organelles operate near maximum capacity during wakefulness, with electron transport chain (ETC) activity generating substantial ROS as obligate byproducts of oxidative phosphorylation [4]. The superoxide anion radical (O₂•⁻), produced primarily at complexes I and III, represents the primary source of mitochondrial oxidative stress, subsequently generating hydrogen peroxide and, through iron-catalyzed Fenton reactions, the highly reactive hydroxyl radical (•OH) [17].

The brain's vulnerability to oxidative damage is compounded by its membrane composition. Neuronal plasma membranes contain high concentrations of PUFAs, particularly docosahexaenoic acid (DHA), which are essential for membrane fluidity and synaptic function but are highly susceptible to lipid peroxidation [3]. Once initiated, lipid peroxidation propagates through chain reactions that can damage extensive membrane regions before termination. The products of lipid peroxidation, including malondialdehyde (MDA) and 4-hydroxynonenal (4-HNE), are themselves toxic and can modify proteins and DNA, amplifying oxidative injury [18].

Paradoxically, the brain maintains relatively modest antioxidant defenses compared to other metabolically active organs. Catalase activity in brain tissue is approximately one-tenth that of the liver, and while glutathione peroxidase is present, total glutathione concentrations are lower than in many peripheral tissues [5]. This disparity between oxidative challenge and antioxidant capacity has been termed the "oxidative stress paradox" of the brain and likely reflects evolutionary trade-offs between metabolic efficiency and protective redundancy [19]. The brain's limited intrinsic antioxidant reserves raise questions about whether environmental factors may have historically supplemented endogenous defenses, a possibility explored later in this paper as part of the proposed hypothesis.

Mitochondria themselves represent both the primary source and the primary target of ROS in neurons. The mitochondrial genome, encoding 13 essential ETC subunits, lacks the protective histones of nuclear DNA and possesses limited repair mechanisms [20]. Mitochondrial DNA mutations accumulate with age as heteroplasmy (the coexistence of normal and mutated mitochondrial DNA within the same cell) increases and are significantly elevated in neurodegenerative diseases, including Alzheimer's disease, where CCO deficiency represents the most consistently identified ETC abnormality [21,22]. This creates a potential vicious cycle: oxidative damage impairs ETC function, which increases electron leakage and superoxide production, which causes further oxidative damage. Notably, many mitochondrial antioxidant systems, including melatonin synthesis capacity and the sirtuin family of deacetylases, are evolutionarily ancient, likely acquired during the endosymbiotic incorporation of α-proteobacteria that gave rise to mitochondria [23]. This evolutionary conservation suggests these systems may have served protective functions over deep evolutionary time.

The temporal dynamics of this oxidative burden deserve consideration. During wakefulness, when neuronal activity and ATP demand are maximal, ROS production is correspondingly elevated. Sleep provides an important window for repair and clearance, during which reduced metabolic demand permits mitochondria to shift from maximal ATP production to maintenance functions [6,24]. The glymphatic system, discovered by Nedergaard, provides an additional sleep-dependent clearance mechanism, facilitating the cerebrospinal fluid-mediated removal of extracellular waste products, including amyloid-beta (Aβ) [25,26].

Modern environmental conditions may have disrupted evolutionarily conserved protective mechanisms. Humans evolved under conditions of substantial daily sunlight exposure, including the NIR component that penetrates tissue and reaches mitochondria. Average solar NIR irradiance during outdoor daylight ranges from approximately 20 to 40 mW/cm² depending on latitude, season, time of day, and atmospheric conditions, with proportionally greater NIR representation at sunrise and sunset when shorter wavelengths are preferentially scattered by the atmosphere [7,8]. However, while the proportion of NIR relative to UV increases at sunrise and sunset, the absolute irradiance at these times is substantially lower than at solar noon, making near-noon exposure more efficient for achieving target PBM fluences [7,8].

Contemporary indoor lifestyles have substantially reduced NIR exposure for many populations compared to historical patterns of outdoor activity [7,8]. If NIR serves as a physiological stimulus for endogenous protective mechanisms, as emerging evidence suggests, the reduction in NIR exposure associated with modern indoor lifestyles could theoretically affect these mechanisms. However, this remains entirely speculative. The apparent increase in age-related neurodegenerative disease prevalence in industrialized societies is primarily attributable to increased longevity and improved diagnostic capability, and no epidemiological evidence supports a causal role for reduced NIR exposure. Whether NIR deficiency has any measurable impact on neurodegeneration risk is an open question that would require carefully controlled epidemiological studies comparing populations with different sunlight exposure patterns while accounting for numerous confounders including lifespan, diet, healthcare access, and genetic background.

The progressive decline in both systemic and local antioxidant capacity with aging further exacerbates neuronal vulnerability. Pineal melatonin production peaks in adolescence and declines throughout adulthood, with particularly steep reductions occurring after age 50 [11]. By late life, nocturnal melatonin concentrations may be less than 20% of young adult values. This decline correlates with increased sleep fragmentation, altered circadian rhythmicity, and reduced antioxidant capacity that characterize normal aging [27]. Similarly, patients with Alzheimer's disease have cerebrospinal fluid melatonin concentrations that are approximately 20% of those seen in age-matched controls, a reduction that appears to begin in preclinical disease stages [12,13]. Whether this melatonin deficit contributes to disease pathogenesis or represents a consequence of neurodegeneration remains under investigation, but the correlation suggests melatonin restoration could provide therapeutic benefit.

A highly useful but frequently overlooked consideration in this framework is substrate availability for downstream antioxidant synthesis. Melatonin-induced upregulation of glutathione-synthesizing enzymes can only produce protective glutathione if adequate precursor amino acids, such as glutamate, cysteine, and glycine, are available. Emerging evidence demonstrates that glycine, traditionally considered non-essential, functions as a conditionally essential amino acid whose endogenous synthesis is insufficient to meet total metabolic demands [28,29]. Aging populations commonly exhibit deficiencies in both glycine and cysteine, and supplementation with these precursors corrects age-related glutathione deficits [30]. This substrate requirement represents a potential rate-limiting step in the proposed protective cascade, with implications for both understanding variable responses to PBM and optimizing therapeutic protocols.

Critical evaluation of brain vulnerability evidence

The evidence establishing the brain's exceptional metabolic vulnerability and oxidative stress susceptibility is robust, derived from multiple independent methodologies including biochemical assays, imaging studies, and post-mortem analyses. The 20% oxygen consumption figure has been replicated across numerous studies using diverse techniques [1]. However, several limitations warrant consideration. First, much of our understanding of neuronal mitochondrial function derives from rodent models, and species differences in metabolic rate, antioxidant capacity, and lifespan may limit direct extrapolation to humans. Second, the relationship between oxidative stress markers and functional outcomes remains correlational in most human studies; whether oxidative damage causes neurodegeneration or represents an epiphenomenon of other pathological processes cannot be definitively resolved from observational data. Third, the environmental mismatch hypothesis, that reduced NIR exposure contributes to neurodegeneration, remains entirely speculative and would require epidemiological studies comparing populations with different sunlight exposure patterns while controlling for numerous confounders.

The proposed NIR radiation-melatonin-glutathione cascade

**Figure 1:**
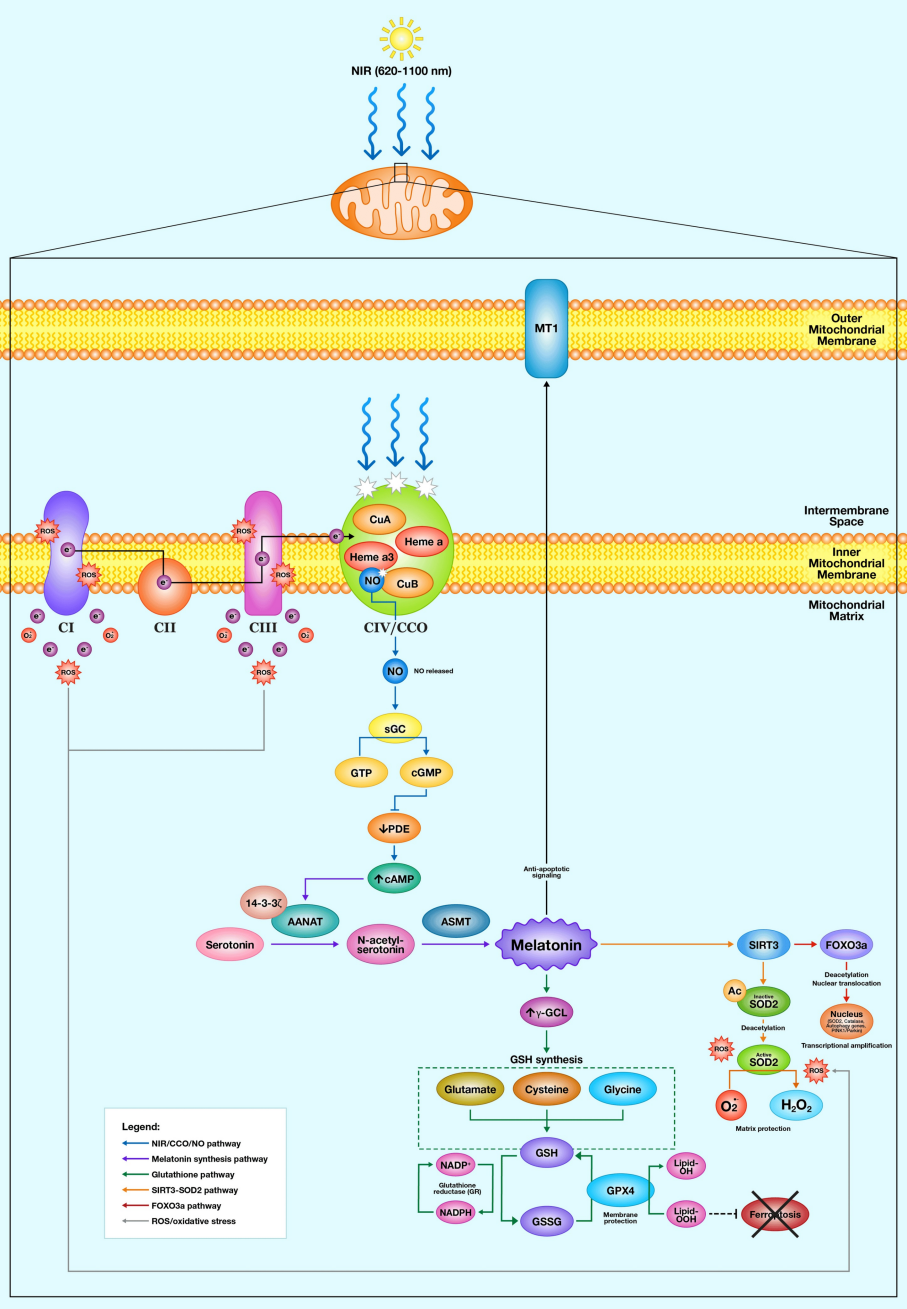
Proposed mechanism of NIR-induced mitochondrial antioxidant protection NIR photons (620-1100 nm) are absorbed by CCO at the inner mitochondrial membrane, photodissociating NO from the CuB/heme a3 binuclear center. Released NO activates sGC, increasing cGMP, which inhibits PDE and elevates mitochondrial cAMP. Elevated cAMP activates AANAT, converting serotonin to melatonin via N-acetylserotonin and ASMT. Locally synthesized melatonin initiates three parallel protective pathways: (1) upregulation of GSH synthesis through γ-GCL and activation of GPX4 for membrane protection; (2) activation of SIRT3, which deacetylates SOD2 for superoxide neutralization; and (3) SIRT3-mediated FOXO3a activation for the transcriptional amplification of antioxidant and autophagy genes. The automitocrine loop (dashed arrow) indicates melatonin action on mitochondrial MT1 receptors. Solid arrows indicate established pathways; dashed arrows indicate hypothesized connections. NIR: near-infrared; CCO: cytochrome c oxidase; NO: nitric oxide; sGC: soluble guanylyl cyclase; PDE: phosphodiesterase; AANAT: arylalkylamine N-acetyltransferase; ASMT: acetylserotonin methyltransferase; GSH: glutathione; γ-GCL: γ-glutamylcysteine ligase; GPX4: glutathione peroxidase 4; SIRT3: sirtuin 3; SOD2: superoxide dismutase 2; FOXO3a: forkhead box O3a; ETC: electron transport chain; ROS: reactive oxygen species; O₂•⁻: superoxide; H₂O₂: hydrogen peroxide; cAMP: cyclic adenosine monophosphate; cGMP: cyclic guanosine monophosphate Image created by the Mercola graphic design team, located in Manila, Philippines, using Adobe Illustrator and Adobe Photoshop (San Jose, California, United States)

The core hypothesis of this paper proposes that NIR activates a protective cascade originating with CCO and culminating in comprehensive mitochondrial antioxidant defense. This section examines each component of the proposed pathway, evaluating the evidence supporting individual steps and acknowledging where direct experimental validation of the complete integrated cascade remains needed. Figure 1 provides an integrated schematic of the proposed mechanistic framework linking NIR radiation to mitochondrial melatonin synthesis and downstream antioxidant defenses.

Because the proposed NIR-mitochondrial melatonin-antioxidant cascade integrates findings from multiple fields with differing levels of experimental support, Table 1 summarizes the relative strength of evidence for each major component of the framework. This hierarchy distinguishes well-established mechanisms from plausible but as-yet untested connections, providing a structured context for the mechanistic sections that follow.

**Table 1:** Evidence hierarchy for components of the proposed NIR-mitochondrial melatonin-antioxidant cascade Evidence categories: ★★★★: well-established with multiple independent replications; ★★★☆: supported by limited studies requiring replication; ★★☆☆: plausible hypothesis awaiting direct testing; ★☆☆☆: speculative prediction/logical extrapolation CCO: cytochrome c oxidase; NIR: near-infrared; NO: nitric oxide; CNS: central nervous system; AANAT: arylalkylamine N-acetyltransferase; ASMT: acetylserotonin methyltransferase; SIRT3: sirtuin 3; SOD2: superoxide dismutase 2; PBM: photobiomodulation; RCTs: randomized controlled trials

Component	Evidence level	Key supporting evidence	Major gaps
CCO as NIR chromophore	★★★★	Action spectrum matching [31,32]; photodissociation studies [33,34]; functional demonstrations [9,10]	In vivo human brain tissue confirmation
NIR photodissociates NO from CCO	★★★★	Isolated mitochondria studies [33]; cell culture validation [34]	Quantitative dose-response in human CNS
Mitochondria contain melatonin synthesis enzymes	★★★★	AANAT/ASMT localization [14]; isotope-labeled synthesis [14,15]	Tissue-specific variation in humans
Mitochondrial melatonin lacks a circadian rhythm	★★★☆	Animal studies [14,15]	Human tissue confirmation
NIR triggers mitochondrial melatonin synthesis	★★☆☆	Theoretical framework [35]; indirect evidence	No direct experimental demonstration
Melatonin upregulates glutathione synthesis	★★★★	Multiple animal and cell studies [36-41]	Human CNS-specific data
Melatonin activates SIRT3-SOD2 axis	★★★☆	Cardiac, intestinal, lung models [42-44]	Brain-specific validation
Glycine/cysteine limit glutathione synthesis	★★★★	Clinical trials [30]; metabolic studies [28,29]	Integration with PBM protocols
PBM improves cognition in dementia	★★★☆	Pilot RCTs [45-47]; case series	Large, rigorous phase III trials
Complete integrated cascade	★☆☆☆	Mechanistic plausibility	Unified experimental validation

CCO as the primary photoreceptor

CCO, the terminal enzyme of the mitochondrial ETC, has been identified as the primary chromophore responsible for PBM effects in the red to NIR spectrum (620-1100 nm) [9,10,31]. CCO contains four redox-active metal centers, specifically two copper (CuA and CuB) and two heme (heme a and heme a3), that absorb photons in this wavelength range [48]. The action spectrum of PBM, determined by comparing biological effects across wavelengths, closely matches the absorption spectrum of oxidized CCO, providing strong correlative evidence for CCO as the responsible chromophore [31,32].

Among proposed signaling pathways, nitric oxide (NO) has been studied most extensively as the link between light absorption at CCO and subsequent cellular signaling. Under physiological conditions, NO binds to the CuB/heme a3 binuclear center of CCO, competitively inhibiting oxygen binding and reducing enzyme activity [33]. Photon absorption at specific NIR wavelengths has been demonstrated to photodissociate NO from CCO, restoring ETC activity and simultaneously releasing NO as a signaling molecule [33,34]. This photodissociation mechanism has been confirmed in isolated mitochondria and cell culture systems, though in vivo confirmation in mammalian brain tissue, while supported by indirect evidence, requires additional direct measurement.

The functional consequences of CCO photostimulation include increased mitochondrial membrane potential, enhanced ATP synthesis, and release of ROS at levels sufficient for signaling but below cytotoxic thresholds [49-51]. Studies using broadband NIR spectroscopy have demonstrated that transcranial NIR application in humans produces measurable increases in oxidized CCO concentration in the underlying cortex, accompanied by increased oxygenated hemoglobin, indicating both direct CCO effects and secondary hemodynamic changes [52,53]. These findings establish that NIR photons reach and interact with brain mitochondria at therapeutically relevant intensities.

It should be noted that some investigators have questioned whether CCO is the sole or even primary photoacceptor for all PBM effects. Alternative chromophores, including mitochondrial-bound water and light-sensitive ion channels, have been proposed [54]. The weight of current evidence, however, supports CCO as a major mediator of NIR effects on mitochondrial function, even if additional mechanisms may contribute.

Critical evaluation of CCO photoreceptor evidence

The evidence supporting CCO as a primary NIR chromophore is among the strongest in the proposed framework, based on convergent findings from spectroscopic studies, functional assays, and human neuroimaging. The action spectrum matching between PBM biological effects and CCO absorption provides compelling correlative support [31,32]. However, several limitations merit consideration. First, most mechanistic studies demonstrating NO photodissociation have been conducted in isolated mitochondria or cell culture systems; the extent to which these findings translate to intact tissue with its complex optical properties and competing chromophores remains incompletely characterized. Second, the quantitative relationship between NIR dose at the scalp surface and CCO activation in deep brain structures has not been precisely established, complicating protocol optimization for transcranial applications. Third, the proposal by Sommer [54] that mitochondrial-bound water rather than CCO serves as the primary photoacceptor has not been definitively refuted, and the relative contributions of multiple chromophores may vary with wavelength and tissue type. Despite these limitations, CCO remains the best-supported candidate for mediating NIR effects on mitochondrial function.

The mitochondrial melatonin synthesis pathway

The discovery that mitochondria possess the enzymatic machinery for melatonin synthesis represents a fundamental advance in understanding extrapineal melatonin biology. Suofu and colleagues demonstrated that mouse brain non-synaptosomal mitochondria contain both AANAT and ASMT localized to the mitochondrial matrix, along with the chaperone 14-3-3ζ that stabilizes AANAT and increases its affinity for the substrate serotonin [14]. When incubated with deuterated serotonin, isolated mitochondria produced high concentrations of deuterated melatonin, providing direct evidence of local synthesis capacity.

Notably, mitochondrial AANAT content did not exhibit the circadian fluctuations characteristic of pineal AANAT, suggesting fundamentally different regulatory mechanisms [14,15]. Pineal melatonin synthesis is controlled by the nocturnal release of norepinephrine from sympathetic nerve terminals, which activates transmembrane adenylyl cyclase (tmAC) and increases cytosolic cyclic adenosine monophosphate (cAMP). Because cAMP is mitochondrial membrane-impermeable, this pathway cannot directly regulate mitochondrial AANAT [23]. The regulatory mechanisms controlling mitochondrial melatonin synthesis remain incompletely characterized but appear to involve mitochondrial soluble adenylyl cyclase (sAC) responding to local signals rather than systemic noradrenergic input.

This is where the proposed cascade becomes more speculative, warranting careful interpretation. Mitochondrial soluble sAC, unlike transmembrane sAC, is directly activated by bicarbonate ions (HCO₃⁻) and calcium rather than G-protein signaling [55,56]. Emerging evidence demonstrates that mitochondrial pools of sAC regulate oxidative phosphorylation, mitochondrial dynamics, and metabolic homeostasis [56]. We hypothesize that NIR photostimulation of CCO enhances ETC activity and Krebs cycle flux, increasing carbon dioxide (CO₂) production. Mitochondrial carbonic anhydrases rapidly convert this CO₂ to bicarbonate, which directly activates sAC to produce cAMP [57]. Furthermore, a 2023 review by Tan and colleagues explicitly proposed that NIR radiation stimulates mitochondrial melatonin synthesis, noting that modern indoor environments have eliminated NIR exposure with potential consequences for local melatonin production [35]. The resulting cAMP-dependent activation of AANAT would, if this pathway operates as proposed, accelerate melatonin synthesis from available serotonin. This proposed NIR→enhanced respiration→bicarbonate/calcium→sAC→cAMP→AANAT pathway is mechanistically plausible, and each individual step is supported by evidence, but the complete cascade as a unified NIR-responsive system has not been directly validated experimentally. Studies tracing this entire pathway in response to NIR stimulation would substantially strengthen the hypothesis.

Regardless of the specific triggering mechanism, the presence of melatonin-synthesizing capacity in all brain cell types, including neurons, astrocytes, and microglia, suggests that mitochondrial melatonin production may be ubiquitous rather than confined to specialized secretory cells [15]. The functional significance is profound: each mitochondrion may be capable of producing its own antioxidant defense, providing protection precisely where ROS are generated. This "automitocrine" model, proposed by Reiter and colleagues, envisions melatonin acting on the same mitochondria that produced it through receptors localized to the mitochondrial outer membrane [36,58].

Recent estimates suggest that the majority of melatonin produced in mammals derives from extrapineal sources, potentially within mitochondria, with the pineal gland contributing a smaller fraction than traditionally assumed [15]. While some researchers have estimated pineal contribution at less than 5% of total body melatonin, this figure is derived from indirect calculations comparing tissue mass and synthetic capacity; direct measurements validating this estimate across different tissues and physiological states remain limited [15]. This represents a paradigm shift from the traditional view of melatonin as primarily a pineal hormone. The implications for understanding melatonin's antioxidant functions are substantial: rather than depending on circulating hormone reaching target tissues, cells may generate protective melatonin on-site in response to local metabolic demands.

Critical evaluation of mitochondrial melatonin synthesis evidence

The discovery of mitochondrial melatonin synthesis machinery represents robust, well-replicated findings from Suofu et al. [14] and subsequent studies [15]. The isotope-labeling experiments providing direct evidence of local synthesis capacity are particularly compelling. However, several important gaps remain. First, most studies have been conducted in rodent tissues, and a comprehensive characterization of mitochondrial melatonin synthesis in human brain tissue is lacking. Second, the regulatory mechanisms controlling mitochondrial AANAT activity remain incompletely understood; the proposed sAC-mediated pathway is plausible but has not been directly demonstrated in response to NIR stimulation. Third, the quantitative contribution of mitochondrial versus pineal melatonin to total body pools under various physiological conditions requires further investigation. The hypothesis that NIR radiation triggers mitochondrial melatonin synthesis, while mechanistically plausible and supported by the theoretical framework of Tan et al. [35], represents the least-established link in the proposed cascade and constitutes a critical priority for experimental validation.

Melatonin-mediated glutathione amplification

The antioxidant actions of melatonin extend substantially beyond its direct radical scavenging capacity. Among the most significant downstream effects is the upregulation of glutathione synthesis and the enzymes of glutathione metabolism. This amplification is particularly important for mitochondria, which, lacking catalase, depend entirely on glutathione peroxidase for hydrogen peroxide detoxification [5].

Melatonin stimulates gamma-glutamylcysteine ligase (γ-GCL), the rate-limiting enzyme in glutathione synthesis, leading to increased intracellular glutathione concentrations [36,37]. Multiple studies have documented melatonin-induced increases in glutathione peroxidase and glutathione reductase activity, accelerating both the reduction of peroxides and the regeneration of reduced glutathione from its oxidized form (GSSG) [38-41]. In isolated rat liver and brain mitochondria challenged with oxidative stress, melatonin at nanomolar concentrations significantly increased glutathione content and the activities of glutathione-related enzymes [41].

The quantitative significance of this amplification is substantial. Studies have reported that melatonin demonstrates considerable efficacy in scavenging peroxyl radicals in vitro, though direct comparisons with other antioxidants depend heavily on experimental conditions [59]. The capacity of one melatonin molecule to stimulate the production of multiple glutathione molecules and activate multiple enzyme systems provides multiplicative protection exceeding what direct scavenging alone could achieve. Furthermore, melatonin metabolites generated during radical scavenging, particularly N1-acetyl-N2-formyl-5-methoxykynuramine (AFMK) and N1-acetyl-5-methoxykynuramine (AMK), retain substantial antioxidant activity, creating what has been termed an "antioxidant cascade" [60].

Critical evaluation of glutathione amplification evidence

The evidence that melatonin upregulates glutathione synthesis and related enzymes is well-established across multiple experimental systems, including cell culture, isolated organelles, and whole animal models [36-41]. Systematic reviews and meta-analyses of animal studies confirm consistent effects on oxidative stress markers [41]. However, translation to human clinical outcomes remains limited. Most human studies of melatonin supplementation have focused on sleep outcomes rather than antioxidant biomarkers, and the extent to which oral melatonin supplementation achieves sufficient tissue concentrations to activate glutathione-related pathways in the central nervous system (CNS) is uncertain. Furthermore, whether locally synthesized mitochondrial melatonin produces the same enzymatic effects as exogenously administered melatonin requires investigation.

Substrate availability: the critical rate-limiting factor

However, melatonin-induced upregulation of glutathione synthesis can only proceed if adequate precursor substrates are available. Glutathione is synthesized from three amino acids: glutamate, cysteine, and glycine. While cysteine availability has traditionally been considered the primary rate-limiting factor, recent evidence demonstrates that glycine may also limit glutathione synthesis under physiological conditions. Tissue glycine concentrations are lower than the Michaelis constant (Km) of glutathione synthase for glycine, meaning that cellular glycine levels may be insufficient to sustain optimal glutathione production, particularly in states of increased oxidative stress [28]. N-acetylcysteine (NAC) effectively serves as a cysteine donor, providing substrate for glutathione synthesis, while glycine must be obtained from either diet or endogenous synthesis [28].

**Figure 2:**
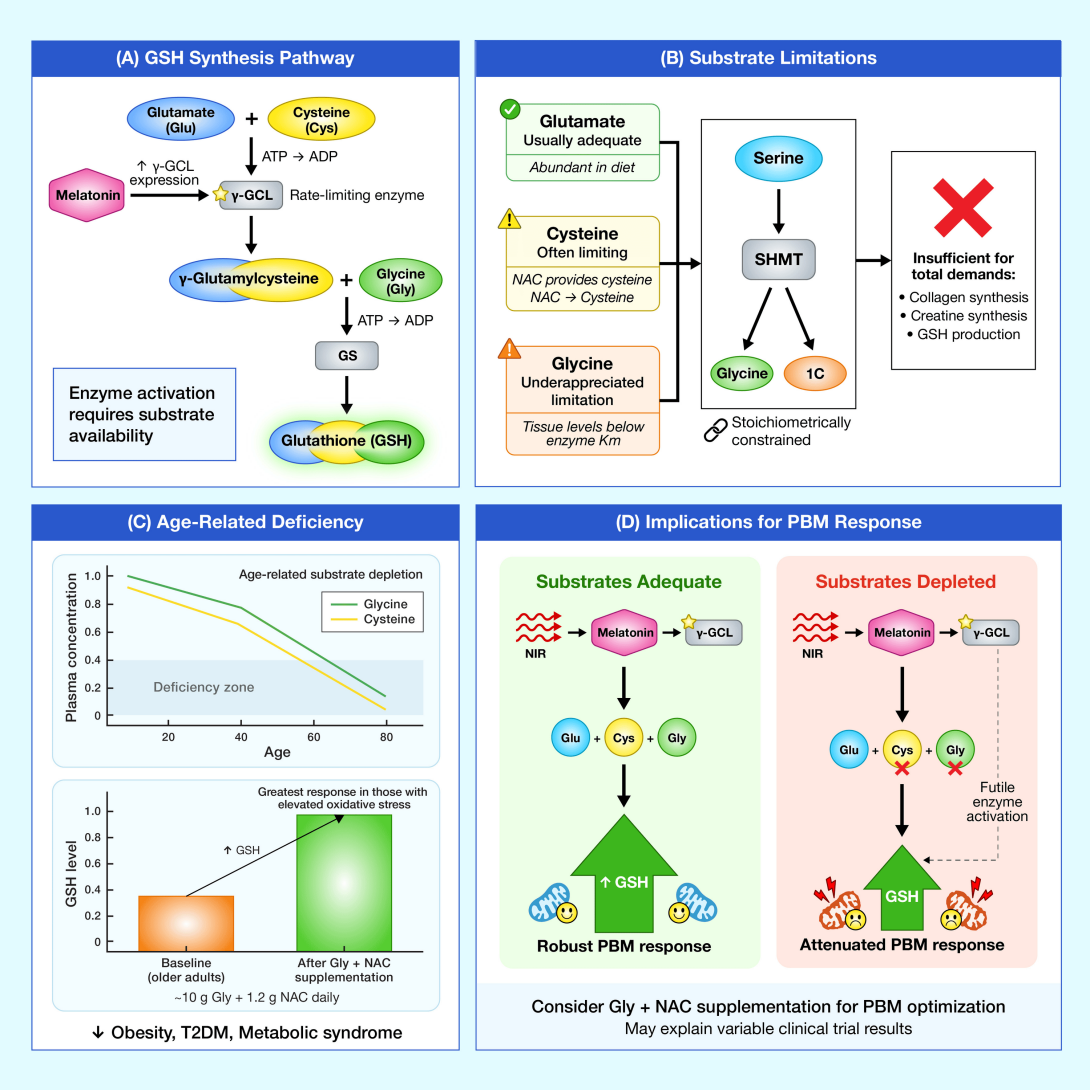
GSH synthesis pathway and substrate availability as a rate-limiting factor (A) The GSH synthesis pathway: GSH is synthesized in two ATP-dependent steps. First, γ-GCL (also called γ-glutamylcysteine synthetase) conjugates glutamate and cysteine to form γ-glutamylcysteine: this is the rate-limiting enzymatic step. Second, GS adds glycine to complete the tripeptide. Melatonin upregulates γ-GCL expression, but enzyme activation can only increase GSH production if substrates are available. (B) Substrate limitations: While cysteine has traditionally been considered the primary rate-limiting substrate, tissue glycine concentrations are typically below the Km of GS, meaning glycine availability may also limit synthesis. Endogenous glycine production from serine via SHMT is insufficient to meet total metabolic demands. (C) Age-related deficiency: Circulating glycine and cysteine levels decline with age, correlating with decreased GSH levels. Clinical trials demonstrate that combined supplementation with glycine and NAC restores GSH in older adults, with the greatest responses in those with elevated baseline oxidative stress. (D) Implications for PBM: Variable responses to PBM may reflect substrate availability; individuals with adequate glycine and cysteine stores may respond robustly to NIR-induced melatonin synthesis, while substrate-depleted individuals show attenuated responses despite identical light parameters. GSH: glutathione; γ-GCL: γ-glutamylcysteine ligase; GS: glutathione synthetase; SHMT: serine hydroxymethyltransferase; NAC: N-acetylcysteine; PBM: photobiomodulation; NIR: near-infrared; Glu: glutamate; Cys: cysteine; Gly: glycine; ATP: adenosine triphosphate Image created by the Mercola graphic design team, located in Manila, Philippines, using Adobe Illustrator and Adobe Photoshop (San Jose, California, United States)

Crucially, glycine should be considered a conditionally essential amino acid. Endogenous glycine biosynthesis from serine is constrained by stoichiometric limitations in the glycine hydroxymethyltransferase reaction, producing insufficient quantities to meet total metabolic demands, including collagen turnover, creatine synthesis, and glutathione production [28,29]. This metabolic constraint becomes particularly significant in aging, when demands for antioxidant defense increase while glycine availability often declines. Lower circulating glycine levels have been consistently observed in association with aging, obesity, type 2 diabetes, and metabolic syndrome [28,29]. Figure 2 outlines the glutathione biosynthetic pathway and emphasizes substrate availability as a critical determinant of antioxidant capacity.

Recent clinical evidence confirms that combined supplementation with glycine and cysteine (with cysteine provided as NAC) corrects age-related glutathione deficiency. In a randomized controlled trial in healthy older adults, Lizzo et al. [30] demonstrated that doses of 4.8-7.2 g/day in a 1:1 glycine-to-NAC ratio produced dose-dependent increases in plasma glycine and cysteine bioavailability within 60 minutes of oral intake. Importantly, individuals with baseline evidence of elevated oxidative stress demonstrated the greatest glutathione increases in response to supplementation, suggesting that substrate deficiency may limit endogenous antioxidant responses precisely in those who need protection most [30]. Based on the totality of evidence, daily supplementation with approximately 10 g of glycine and 1.2 g of NAC (600 mg twice daily) appears sufficient to ensure adequate substrate for glutathione synthesis without risking reductive stress from excessive antioxidant supplementation [28,29].

The implications for the proposed NIR-melatonin-glutathione framework are significant. If NIR-induced mitochondrial melatonin synthesis activates glutathione-synthesizing enzymes, the protective effect will be constrained by substrate availability. In substrate-depleted states common in aging, the cascade may fail to achieve its full protective potential regardless of adequate NIR stimulation or melatonin signaling. This represents a potential explanation for variable responses to PBM observed in clinical studies: individuals with adequate glycine and cysteine stores may respond robustly, while those with substrate deficiency may show attenuated or absent responses despite identical light exposure parameters.

The interaction between melatonin and glutathione also involves synergistic effects. Melatonin can regenerate glutathione from GSSG, and glutathione in turn may protect melatonin from oxidative degradation. These reciprocal interactions create a mutually reinforcing antioxidant network within mitochondria.

Critical evaluation of substrate availability evidence

The evidence that glycine and cysteine availability can limit glutathione synthesis is well-supported by metabolic studies [28,29] and clinical trials demonstrating that supplementation corrects age-related deficits [30]. This represents one of the more actionable components of the proposed framework. However, the hypothesis that substrate availability explains variable responses to PBM has not been directly tested. Clinical trials comparing PBM outcomes in substrate-replete versus substrate-depleted individuals, or comparing PBM alone versus PBM combined with glycine/NAC supplementation, would provide critical evidence for this proposed mechanism.

Coordinated mitochondrial defense: the SIRT3-SOD2 and glutathione-glutathione peroxidase 4 (GPX4) axes

**Figure 3:**
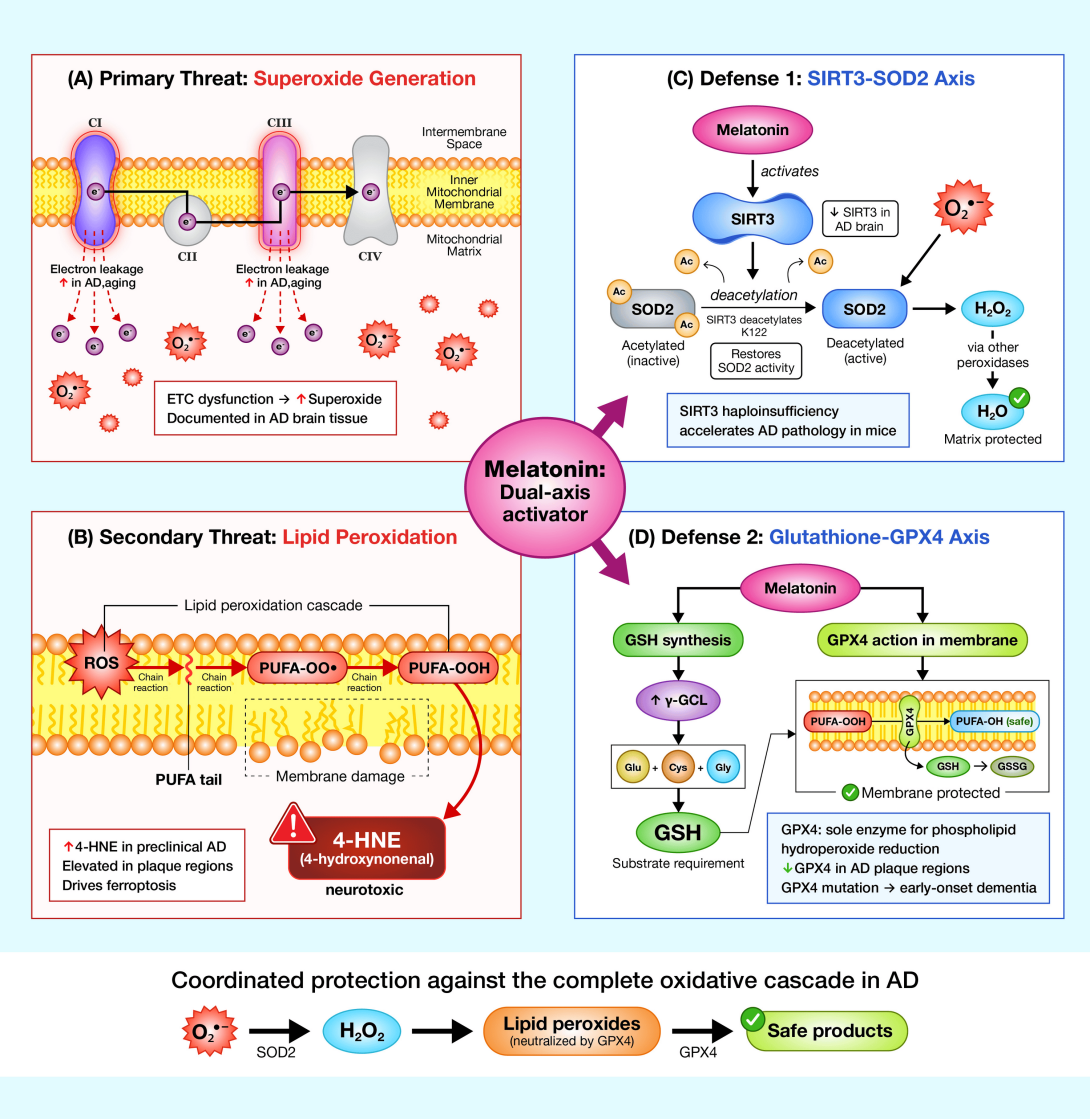
Dual oxidative threats in AD and coordinated mitochondrial defense mechanisms Mitochondrial dysfunction in AD produces two distinct but interconnected oxidative insults. (A) Primary threat: ETC dysfunction at complexes I and III increases electron leakage, generating excessive O₂•⁻ in the matrix. (B) Secondary threat: Superoxide-derived reactive oxygen species initiate lipid peroxidation in mitochondrial membranes, producing neurotoxic aldehydes including 4-HNE that propagate through chain reactions. (C) The SIRT3-SOD2 axis: Melatonin activates SIRT3, which deacetylates SOD2 at lysine residues, restoring full enzymatic activity for superoxide dismutation to hydrogen peroxide. (D) The glutathione-GPX4 axis: Melatonin upregulates glutathione synthesis and GPX4 expression; GPX4 is the sole enzyme capable of reducing phospholipid hydroperoxides directly within membranes, preventing ferroptotic propagation. Both defensive systems are compromised in AD brain tissue, and both are restored by melatonin through SIRT3-dependent and Nrf2-dependent mechanisms. The convergence of these pathways on AD pathology, with documented GPX4 reduction and 4-HNE elevation in plaque-associated regions, supports the therapeutic rationale for NIR-induced melatonin synthesis. AD: Alzheimer's disease; ETC: electron transport chain; O₂•⁻: superoxide; 4-HNE: 4-hydroxynonenal; SIRT3: sirtuin 3; SOD2: superoxide dismutase 2; GPX4: glutathione peroxidase 4; NIR: near-infrared; Nrf2: nuclear factor erythroid 2-related factor 2; PUFA: polyunsaturated fatty acid; GSH: reduced glutathione; GSSG: oxidized glutathione Image created by the Mercola graphic design team, located in Manila, Philippines, using Adobe Illustrator and Adobe Photoshop (San Jose, California, United States)

Alzheimer's disease pathology involves dual oxidative insults at the mitochondrial level: primary superoxide generation from ETC dysfunction and secondary membrane lipid peroxidation producing neurotoxic aldehydes including 4-HNE. NIR-induced mitochondrial melatonin synthesis may provide coordinated protection against both pathological arms through the parallel activation of the SIRT3-SOD2 pathway and glutathione-dependent GPX4. Figure 3 summarizes the two principal oxidative challenges characteristic of Alzheimer's disease, namely, mitochondrial superoxide overproduction and lipid peroxidation, and illustrates how melatonin-dependent activation of the SIRT3-SOD2 axis and the glutathione-GPX4 system provides coordinated protection against these complementary injury pathways.

The SIRT3-SOD2 axis: neutralizing superoxide at the source

SIRT3, the primary mitochondrial nicotinamide adenine dinucleotide (NAD⁺)-dependent deacetylase, directly regulates the activity of manganese SOD2, the principal matrix antioxidant enzyme. SIRT3 deacetylates SOD2 at specific lysine residues, which activates the enzyme and enhances its capacity to dismutate superoxide radicals to hydrogen peroxide [58,61]. Multiple studies confirm that melatonin upregulates SIRT3 expression and activity across diverse tissue models, subsequently increasing SOD2 deacetylation and function. In cardiomyocytes subjected to ischemia-reperfusion injury, Yu et al. [42] demonstrated that melatonin treatment increased SIRT3 expression and activity, leading to SOD2 deacetylation, reduced ROS generation, and improved cell survival. Similar findings have been reported in models of sepsis-induced intestinal injury [43] and acute lung injury, where Ning et al. [44] showed that mitochondrial quality control of lung epithelial cells was preserved through the SIRT3-dependent deacetylation of SOD2.

Importantly, SIRT3 deficiency has been associated with Alzheimer's disease pathogenesis in experimental models. Cheng and colleagues demonstrated that SIRT3 haploinsufficiency in APP/PS1 mice accelerates GABAergic interneuron loss and neuronal network hyperexcitability, with premature mortality before five months of age [62]. Ying et al. [63] showed that SIRT3 levels are reduced in association with cerebral cortical Aβ pathology in human Alzheimer's disease patients, with SIRT3 regulating neuronal excitability in an oxidative stress-dependent manner. Tyagi and colleagues further demonstrated that SIRT3 gene deletion in APP/PS1 mice significantly decreased insulin-degrading enzyme (IDE) levels, impairing Aβ degradation [64]. These findings position SIRT3 as a notable node in Alzheimer's disease pathophysiology and suggest that NIR-induced melatonin activation of this pathway could provide disease-relevant neuroprotection.

The glutathione-GPX4 axis: membrane protection against lipid peroxidation

While SOD2 addresses superoxide in the matrix, the GPX4 system provides essential protection against lipid peroxidation in mitochondrial membranes. GPX4 is the sole enzyme capable of reducing complex phospholipid hydroperoxides directly within membrane structures, preventing the accumulation of toxic lipid peroxides that drive ferroptotic cell death [65].

Melatonin enhances this protective axis through multiple mechanisms: upregulation of GCL (the rate-limiting enzyme for glutathione synthesis), increased glutathione reductase activity, and direct enhancement of GPX4 expression through the activation of the nuclear factor erythroid 2-related factor 2 (Nrf2) pathway [65-67]. A comprehensive review by Chuffa and colleagues confirms that melatonin upregulates GPX4, thereby inhibiting the accumulation of toxic lipid peroxidation products, including 4-HNE, and preventing ferroptotic cell death [68].

Convergence of oxidative defense mechanisms on Alzheimer's disease pathology

Both defensive arms, the SIRT3-SOD2 axis and the glutathione-GPX4 system, are compromised in Alzheimer's disease brain tissue. Bradley et al. [69,70] documented elevated 4-HNE levels in vulnerable brain regions beginning in preclinical Alzheimer's disease (PCAD) and persisting through late-stage disease. A 2024 study by Majerníková and colleagues demonstrated that progression of Alzheimer's disease pathology is accompanied by decreased GPX4 expression and increased 4-HNE accumulation specifically in amyloid plaque-associated regions, directly linking ferroptosis mechanisms to Aβ pathology [71]. Thorwald and colleagues confirmed that Alzheimer's disease brains show decreased antioxidant enzymes, including those mediated by glutathione, with greater oxidative damage in lipid rafts, the precise sites of amyloid processing [72].

Most compellingly, Lorenz and colleagues demonstrated that a single point mutation in GPX4 (R152H) that impairs its membrane-protective function is sufficient to cause severe early-onset dementia in children, with proteomic changes strikingly similar to those observed in Alzheimer's disease [73]. This provides the first molecular evidence that ferroptosis can directly drive neurodegeneration in the human brain.

Mechanistic integration: NIR as coordinated activator

If validated, the proposed NIR-induced mitochondrial melatonin synthesis pathway would address the full oxidative cascade implicated in Alzheimer's disease: SIRT3 activation enhances SOD2-mediated superoxide clearance at the ETC, while simultaneous glutathione upregulation and GPX4 activation prevent the downstream propagation of lipid peroxidation. This coordinated response may explain why interventions targeting only single antioxidant pathways have shown limited efficacy in Alzheimer's disease trials, while the multi-arm activation provided by melatonin, and potentially by NIR-induced local melatonin synthesis, offers more comprehensive protection.

The evolutionary conservation of both melatonin and sirtuins, both likely acquired by eukaryotes after the endosymbiotic incorporation of α-proteobacteria, suggests these molecules may have coevolved functionally [23]. The consistent finding across multiple tissue types and injury models that melatonin requires SIRT3 for its mitochondrial protective effects points to a fundamental and conserved interaction. This SIRT3-SOD2 axis, combined with the glutathione-GPX4 pathway, provides comprehensive protection against the major forms of mitochondrial oxidative stress.

Critical evaluation of SIRT3-SOD2 and GPX4 evidence

The evidence linking melatonin to SIRT3-SOD2 activation is consistent across multiple tissue models, including cardiac [42], intestinal [43], and pulmonary [44] systems. The association between SIRT3 deficiency and Alzheimer's disease pathology in animal models [62-64] is well-documented. However, several limitations warrant consideration. First, most melatonin-SIRT3 studies have been conducted in non-neuronal tissues; brain-specific validation is limited. Second, the studies demonstrating SIRT3's role in Alzheimer's disease have primarily used transgenic mouse models, which imperfectly recapitulate human disease. Third, the critical link, that NIR-induced mitochondrial melatonin synthesis activates SIRT3 in brain tissue, has not been directly demonstrated. In contrast, the GPX4-ferroptosis connection to neurodegeneration is strongly supported by the human genetic evidence from Lorenz et al. [73], providing compelling proof-of-concept that this pathway can drive dementia. Overall, the individual components are well-supported, but integration within the NIR-responsive framework requires experimental validation.

The SIRT3-FOXO3a longevity axis

**Figure 4:**
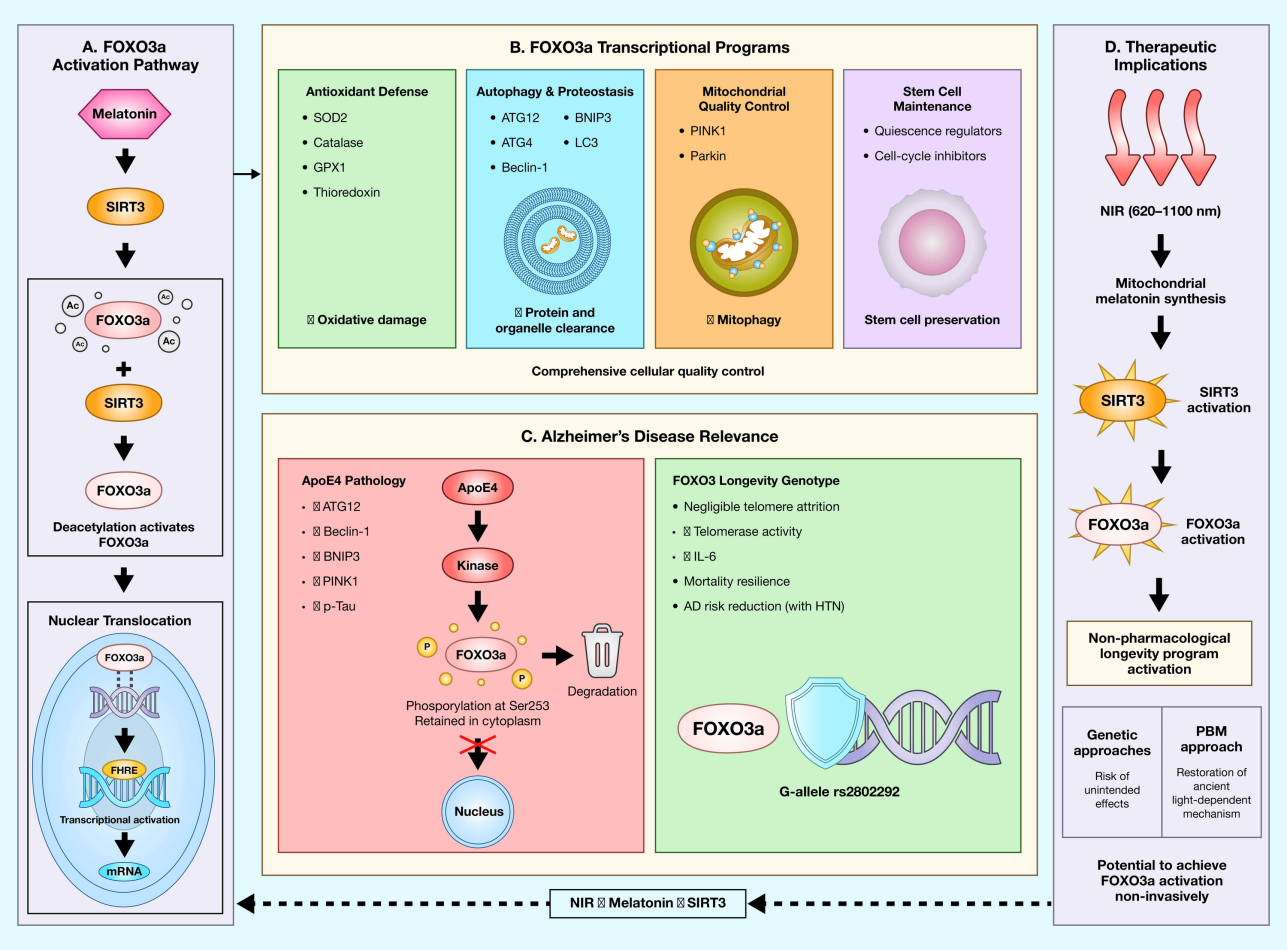
The SIRT3-FOXO3a longevity axis and its integration with NIR-induced melatonin synthesis (A) FOXO3a activation pathway: Melatonin stimulates SIRT3, which deacetylates FOXO3a at multiple lysine residues. Deacetylated FOXO3a translocates to the nucleus where it binds to FHREs in target gene promoters. (B) FOXO3a transcriptional targets encompass four major cellular quality control programs: antioxidant defense (SOD2, catalase, GPX1), autophagy and proteostasis (ATG genes, BNIP3, LC3), mitochondrial quality control (PINK1, Parkin for mitophagy), and stem cell maintenance (quiescence regulators). (C) Relevance to AD: ApoE4, the major AD genetic risk factor, represses FOXO3a through increased phosphorylation at Ser253, leading to cytoplasmic retention and degradation. This results in decreased autophagy-related proteins (ATG12, Beclin-1, BNIP3, PINK1) and correlates with increased phosphorylated tau levels. The FOXO3 longevity genotype (G-allele of rs2802292) confers protection against AD risk, particularly in the context of hypertension. (D) Implications: If NIR-induced melatonin synthesis activates SIRT3-FOXO3a signaling, PBM could provide non-pharmacological activation of one of evolution's most validated longevity programs. SIRT3: sirtuin 3; FOXO3a: forkhead box O3a; NIR: near-infrared; FHREs: forkhead response elements; SOD2: superoxide dismutase 2; AD: Alzheimer's disease; PBM: photobiomodulation; Ac: acetyl group; P: phosphate group Image created by the Mercola graphic design team, located in Manila, Philippines, using Adobe Illustrator and Adobe Photoshop (San Jose, California, United States)

Beyond its effects on glutathione metabolism and SOD2 activation, melatonin activates a coordinated mitochondrial defense program centered on SIRT3 and its important downstream target, the transcription factor forkhead box O3a (FOXO3a). This pathway represents not merely a complementary arm of melatonin-mediated mitochondrial protection but also, potentially, the most significant longevity-promoting mechanism within the entire proposed cascade. Figure 4 depicts the SIRT3-FOXO3a signaling axis and its proposed integration within the NIR-induced mitochondrial melatonin framework.

FOXO3 as the premier human longevity gene

FOXO3 has emerged as arguably the most validated longevity gene in humans, with genetic associations now confirmed across Japanese, German, Italian, Danish, Chinese, and American populations [74]. The longevity-associated G-allele of FOXO3 SNP rs2802292 confers substantial protection against age-related mortality, with the effect increasing at more extreme ages [74]. Unlike most genetic associations with complex traits, the FOXO3-longevity association has proven remarkably robust, replicating across ethnic groups and study designs with consistent effect directions [74,75]. Table 2 compiles cross-population genetic studies demonstrating consistent associations between FOXO3 variants and human longevity, including evidence linking FOXO3 to resilience against age-related disease and Alzheimer's disease risk.

**Table 2:** Cross-population evidence for FOXO3 as a human longevity gene The FOXO3-longevity association has been replicated across diverse ethnic populations with remarkable consistency, establishing FOXO3 as arguably the most validated longevity gene in humans. FOXO3: forkhead box O3; OR: odds ratio; AD: Alzheimer's disease; GWAS: genome-wide association studies

Population	Key finding	Reference
Japanese-American	First GWAS identifying FOXO3 longevity association; OR 1.5-2.75 for reaching advanced ages	Willcox et al., as reviewed in [74]
German centenarians	Replication of longevity association in the European population	Flachsbart et al., as reviewed in [74]
Italian centenarians	Independent confirmation in the Southern European population	As reviewed in [74]
Danish nonagenarians	Confirmation in the Northern European population	As reviewed in [74]
Chinese Han population	Replication in the East Asian population	As reviewed in [74]
American populations	Multi-ethnic confirmation	As reviewed in [74]
Okinawan Japanese	G-allele carriers show negligible telomere attrition with age; higher telomerase activity	Torigoe et al. [75]
Multi-ethnic (Kuakini)	Proteomic basis of "mortality resilience" identified; 44 stress proteins modulated	Donlon et al. [76]
Men with late-life hypertension	FOXO3 longevity genotype ameliorates AD incidence	Chen et al. [77]
Elderly with hypertension	FOXO3 genotype attenuates cerebral microinfarct risk	Nakagawa et al. [78]

Mechanistically, the longevity-associated FOXO3 variants appear to confer "mortality resilience", the capacity to withstand and recover from aging-related stressors that would otherwise prove fatal [76]. Proteomic analyses by Donlon et al. [76] identified 44 stress proteins whose mortality-increasing effects are dampened by the FOXO3 longevity genotype, with biological pathways including innate immunity, bone morphogenetic protein signaling, leukocyte migration, and growth factor response. Consistent with these findings, a 2023 review characterized FOXO3 as a central transcriptional integrator of stress-responsive pathways governing cellular maintenance and aging [79].

Recent studies have revealed striking effects of the FOXO3 longevity genotype on cellular aging mechanisms. In Okinawan Japanese populations, renowned for exceptional longevity, Torigoe et al. [75] demonstrated that carriers of the FOXO3 G-allele show negligible telomere attrition with age, in contrast to the progressive telomere shortening observed in non-carriers. This protection against telomere shortening was accompanied by higher telomerase activity and modestly lower levels of pro-inflammatory interleukin-6 (IL-6) [75]. These findings suggest FOXO3 may preserve replicative capacity in stem cell populations throughout the lifespan.

FOXO3a functions in cellular quality control

The functional diversity of FOXO3a explains its outsized impact on healthspan and longevity.

Antioxidant defense: FOXO3a directly binds to and transactivates the promoters of key antioxidant genes including SOD2 and catalase [80]. SIRT3-mediated deacetylation of FOXO3a promotes its nuclear translocation and DNA binding, amplifying the transcription of these enzymes and providing comprehensive protection against both superoxide and hydrogen peroxide.

Autophagy and proteostasis: FOXO3a directly regulates a network of autophagy genes essential for clearing damaged proteins and organelles. A 2024 review by Xi et al. [81] confirmed that the SIRT3-FOXO3a axis regulates autophagy through the deacetylation of FOXO3a, which then activates E3 ligases Pink1 and Parkin to initiate mitochondrial autophagy. Loss of FOXO function leads to the accumulation of protein aggregates, a hallmark of neurodegenerative disease. Liu et al. [82] established that the AMPK/FOXO3a axis governs autophagy machinery activation, with SIRT3/FOXO3a signaling cascade activation providing neuroprotection in multiple disease models.

Stem cell maintenance: The autophagy-lysosome pathway controlled by FOXO3a is essential for adult neural stem cells to maintain quiescence and re-enter the cell cycle appropriately [82,83]. More broadly, FOXO3a is essential for the long-term maintenance of both hematopoietic stem cells (HSCs) and neural stem cells (NSCs). A 2024 comprehensive review by Zhao et al. [84] on FOXO proteins and stem cell fate demonstrated that FOXOs preserve stem cell pools by activating autophagy networks, coordinating metabolic programs, and counteracting oxidative stress that would otherwise drive stem cells out of quiescence. In aging, reduced autophagic activity correlates with the upregulation of compensatory amino acid transporters, eventually diminishing stem cell populations and regenerative potential [82].

Vascular protection: FOXO3 polymorphisms correlate with lower-than-average morbidity from cardiovascular diseases in long-lived people [74]. Chang et al. [74] established in a 2021 comprehensive review that FOXO3 inactivation is implicated in atherosclerosis, vascular calcification, hypertension, and vascular aging-related diseases affecting the heart, kidney, and cerebrovascular systems. Experimental studies further show that FOXO3-engineered human embryonic stem cell-derived vascular cells improve vascular homeostasis and delay vascular aging [74].

The SIRT3-FOXO3a-melatonin connection

SIRT3 is the primary mitochondrial deacetylase responsible for activating FOXO3a [42-44,62-64]. Experimental studies show that melatonin promotes SIRT3-mediated deacetylation and activation of FOXO3a, an effect confirmed in a 2024 review by Galvani et al. [85] demonstrating that melatonin's mitochondrial actions are mediated through SIRT1 and SIRT3 signaling and downstream FOXO transcription factors. In models of cerebral ischemia-reperfusion injury, Liu et al. [86] showed that melatonin upregulates SIRT3 expression and improves neurological outcomes, with protective effects abolished by SIRT3 inhibition.

The neuroprotective outcomes observed in stroke and traumatic brain injury contexts are intricately tied to the interplay between SIRT3 and the FOXO3a/SOD2 pathway [86]. In Parkinson's disease models, the SIRT3/FOXO3a signaling axis, activated by interventions such as creatine supplementation, promotes antioxidative effects by enhancing the expression of antioxidant enzymes, mitigating oxidative stress and neuroinflammation, and reducing alpha-synuclein oligomerization [81]. The antiepileptic medication perampanel has shown efficacy in inhibiting neuronal damage following subarachnoid hemorrhage by targeting the SIRT3/FOXO3a signaling cascade [81].

Relevance to Alzheimer's disease

The FOXO3a pathway has particular relevance to Alzheimer's disease pathophysiology. ApoE4, the major genetic risk factor for late-onset AD, attenuates autophagy via FOXO3a repression in human brain tissue [87]. Sohn et al. [87] demonstrated that compared to non-carriers, ApoE4 carriers show significantly reduced FOXO3a expression and increased FOXO3a phosphorylation at Ser253 (indicating inactivation), with consequent decreases in autophagy-related proteins including ATG12, Beclin-1, BNIP3, and PINK1. Protein levels of phosphorylated tau were negatively correlated with FOXO3a levels, suggesting a direct link between FOXO3a deficiency and tau pathology [87].

Recent longitudinal data from Chen et al. [77] found that the FOXO3 longevity genotype modifies the relationship between late-life hypertension and Alzheimer's disease risk, with protective effects observed in G-allele carriers. Additionally, Nakagawa et al. [78] demonstrated that the FOXO3 longevity genotype attenuates the impact of hypertension on cerebral microinfarct risk, suggesting the protection of cerebrovascular integrity. A 2024 comprehensive review in Molecular Psychiatry by Zhang et al. [27] identified melatonin as a potential therapeutic agent for Alzheimer's disease through multiple mechanisms including effects on the SIRT-FOXO pathway.

Implications for the NIR-melatonin framework

If NIR-induced mitochondrial melatonin synthesis activates SIRT3 and subsequently FOXO3a, the protective effects would extend far beyond immediate antioxidant defense to encompass autophagy, stem cell preservation, mitochondrial quality control, aggregate clearance, and vascular protection. This represents a potential non-pharmacological, non-genetic approach to activating one of evolution's most powerful longevity programs.

Notably, researchers have proposed genetic engineering approaches to increase FOXO3a expression as an anti-aging intervention. However, such approaches carry risks of unintended consequences and remain technically challenging for clinical application. If the NIR-melatonin-SIRT3-FOXO3a pathway can be validated, PBM would offer a non-invasive means of achieving similar FOXO3a activation through the restoration of an evolutionarily ancient, light-dependent mechanism.

Critical evaluation of FOXO3a evidence

The genetic evidence linking FOXO3 variants to human longevity is among the most robust in aging research, with replication across multiple ethnic populations [74,75]. The mechanistic studies demonstrating FOXO3a's roles in antioxidant defense, autophagy, and stem cell maintenance are well-established [80-84]. The connection between ApoE4 and FOXO3a repression in Alzheimer's disease [78] provides an intriguing mechanistic link. However, the critical gap remains the same: whether NIR-induced melatonin synthesis can activate this pathway in human brain tissue has not been demonstrated. The therapeutic implications are substantial if validated, but the chain of inference from NIR exposure to FOXO3a activation involves multiple untested steps.

The automitocrine hypothesis and mitochondrial melatonin receptors

An intriguing component of this mechanistic framework is the concept of automitocrine signaling, wherein melatonin produced within mitochondria acts on the same organelles that produced it. This model, developed primarily by Reiter and colleagues, proposes that melatonin functions not only as a direct antioxidant but also as a local signaling molecule modulating mitochondrial physiology [35,58,83].

Supporting this concept, Somalo-Barranco and colleagues reported the presence of the MT1 melatonin receptor on mitochondrial membranes in brain tissue [88]. Suofu and colleagues confirmed this localization and demonstrated that mitochondrial MT1 activation inhibits calcium-induced cytochrome c release, a key step in the intrinsic apoptotic pathway [14]. These findings suggest that locally produced melatonin can provide anti-apoptotic protection through receptor-mediated mechanisms in addition to direct antioxidant effects.

Critical evaluation of the automitocrine hypothesis

The automitocrine hypothesis is mechanistically compelling but remains supported by limited experimental evidence. Reports describing MT1 melatonin receptor localization to mitochondrial membranes [14,88] require independent replication, and technical challenges inherent to subcellular fractionation leave open the possibility of contamination from other membrane compartments. As a result, definitive mitochondrial receptor localization has not yet been conclusively established. This uncertainty positions the automitocrine model as one of the more speculative components of the proposed framework. Nevertheless, if confirmed, the concept of mitochondria as sites of hormone synthesis, receptor expression, and autocrine regulation would represent a substantial expansion of the current understanding of mitochondrial biology, extending their role beyond energy metabolism to active participation in cellular survival signaling.

The circadian dimension: timing and the dual melatonin system

Humans evolved under conditions of regular solar exposure, including substantial NIR radiation. From a practical standpoint, the biologically relevant "PBM window" of sunlight (approximately 600-1100 nm, spanning deep red through IR-A) varies dramatically with latitude, season, time of day, and cloud conditions [7,8,89]. The highest dose rates occur near solar noon, when atmospheric path length is shortest; even a few hours away from noon, effective irradiance falls quickly, and the time needed to reach a PBM-like fluence rises nonlinearly [7,89]. Cloud cover further reduces delivery in a wavelength-dependent manner, typically suppressing the longer end of the NIR spectrum more than deep red, such that overcast days can require several-fold longer exposures to achieve similar cumulative NIR fluence [89,90]. As a rule of thumb for an exposed skin patch, midday summer conditions at lower latitudes may require only minutes to approach commonly used PBM fluences, whereas winter and higher latitudes shift the same target into the tens-of-minutes range, and overcast winter conditions can render meaningful dosing impractical outside a narrow midday window [7,8,89].

Because PBM responses are biphasic, the goal is not maximal exposure but repeatable, modest dosing that stays within an effective range [91-93]. In real-world outdoor conditions, the effective delivered dose to tissue is often far lower than "horizontal irradiance" estimates because geometry matters: a skin surface only receives full power when it faces the sun, and movement, body curvature, and partial shading can reduce dose substantially [7,89]. Clothing introduces additional uncertainty, as fabric thickness, weave density, color, and layering variably attenuate red/NIR transmission; consequently, bare-skin exposure is the most reliable way to achieve predictable dosing [7,8]. These considerations support a pragmatic approach for translational use: brief, near-noon exposures on uncovered skin when conditions permit; longer exposures only when latitude/season/clouds lower dose rate; and careful avoidance of stacking multiple high-intensity NIR sources on the same day when the intent is to remain within a targeted PBM fluence window [7,8,89,91-93].

These environmental variables imply that "time outdoors" is a poor proxy for dose; PBM-range sunlight exposure is better conceptualized as a target fluence achieved under conditions that can differ by an order of magnitude day-to-day [7,8,89]. Modern indoor environments have essentially eliminated this exposure pattern. Artificial lighting, even when attempting to replicate visible spectrum characteristics, provides negligible NIR. The consequences for NIR-dependent physiological processes are unknown but potentially significant. If mitochondrial melatonin synthesis is indeed NIR-responsive, contemporary humans may experience chronic deficiency in this locally produced antioxidant, representing a novel form of environmental mismatch.

Time-of-day effects on PBM efficacy

Emerging evidence suggests that PBM efficacy varies with time of day, consistent with the circadian modulation of mitochondrial responsiveness. Shinhmar and colleagues demonstrated that 670 nm light improved color contrast sensitivity in aging humans when applied in the morning (8-9 AM) but not when applied in the afternoon (12-1 PM), with improvements lasting approximately one week [94]. Earlier animal studies from the same laboratory had shown similar time-dependent variation in mitochondrial responses to PBM, with morning applications more effective than evening applications [95].

CCO activity itself exhibits circadian variation. Isobe et al. demonstrated that mitochondrial CCO activity in the suprachiasmatic nucleus was higher during the light phase than during the dark phase in rats, correlating with diurnal patterns of mitochondrial membrane potential and metabolic activity [96]. This suggests that optimal PBM timing may align with periods of peak CCO responsiveness. Thus, the timing of PBM treatments may be an underappreciated parameter affecting therapeutic outcomes. However, it must be acknowledged that most evidence for circadian effects comes from animal models or specific tissues such as the retina and generalizability to whole-brain neuroprotection in humans requires investigation.

The dual melatonin system hypothesis

**Figure 5:**
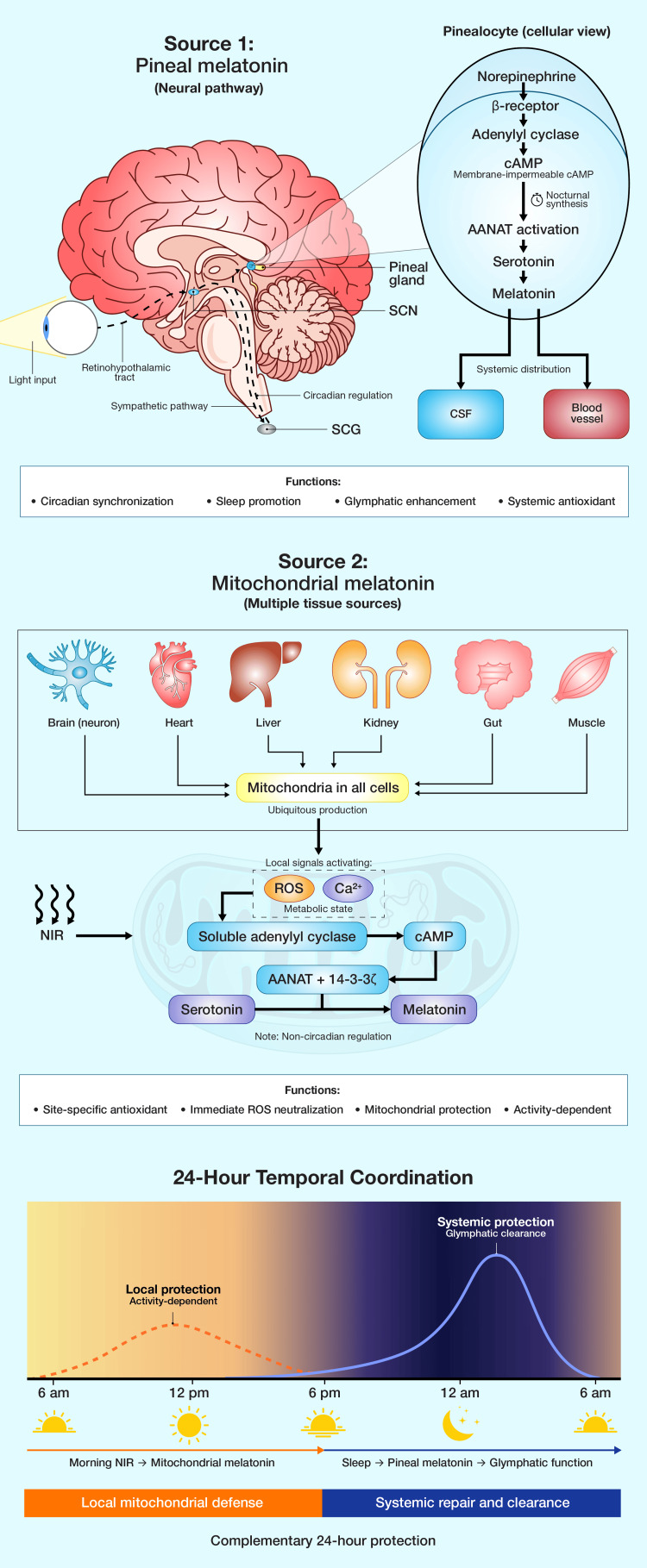
Conceptual framework distinguishing two functionally distinct melatonin systems (A) Source 1 (pineal) melatonin: Synthesized in pinealocytes under noradrenergic control following circadian signals from the SCN. Nocturnal release into CSF and systemic circulation provides chronobiological signaling and supports sleep-dependent processes including glymphatic clearance. Synthesis is regulated by membrane-impermeable cAMP through the classical β-adrenergic pathway. (B) Source 2 (mitochondrial) melatonin: Synthesized locally within mitochondria across all tissue types in response to metabolic signals, potentially including NIR radiation. Regulated by mitochondrial soluble adenylyl cyclase responding to local cues rather than systemic noradrenergic input. Does not exhibit circadian rhythmicity. Provides site-specific antioxidant protection precisely where reactive oxygen species are generated. (C) Temporal coordination across the 24-hour cycle: Daytime NIR exposure may stimulate mitochondrial melatonin for immediate local protection during periods of high metabolic activity, while nighttime pineal melatonin provides systemic support for repair and clearance processes. The two systems are proposed as complementary rather than redundant, together providing continuous protection through mechanistically distinct pathways. SCN: suprachiasmatic nucleus; CSF: cerebrospinal fluid; cAMP: cyclic adenosine monophosphate; NIR: near-infrared Image created by the Mercola graphic design team, located in Manila, Philippines, using Adobe Illustrator and Adobe Photoshop (San Jose, California, United States)

I propose a conceptual framework distinguishing two functionally distinct melatonin systems. Source 1 melatonin, produced by the pineal gland in a circadian fashion, is released into the cerebrospinal fluid and blood, providing systemic chronobiological signals and supporting sleep-associated repair processes including glymphatic clearance. Source 2 melatonin, produced in mitochondria throughout the body in response to local signals potentially including NIR, provides site-specific antioxidant protection without circadian rhythmicity [15]. Figure 5 presents a conceptual model distinguishing pineal-derived circadian melatonin from locally synthesized mitochondrial melatonin.

If this framework is correct, daytime NIR exposure would trigger subcellular melatonin synthesis for immediate local protection, while nighttime pineal melatonin would circulate systemically to support glymphatic function and circadian coordination. These systems would be complementary rather than redundant, providing continuous but mechanistically distinct protection across the 24-hour cycle. This dual-system model integrates established findings regarding pineal melatonin's circadian role and extrapineal melatonin synthesis capacity, though the integrated framework as presented represents a synthesis requiring explicit experimental testing.

Critical evaluation of the circadian and dual melatonin system evidence

The evidence for time-of-day effects on PBM comes primarily from the work of Shinhmar et al. [94] and related studies [95,96]. While intriguing, these findings derive from limited studies in specific tissues (retina, suprachiasmatic nucleus) and require replication and extension to broader CNS applications. The dual melatonin system hypothesis represents a conceptual framework integrating established findings (pineal circadian melatonin, extrapineal synthesis capacity) into a novel synthesis that has not been directly tested experimentally.

The biphasic dose-response relationship: the Goldilocks principle

One of the most consistent findings in PBM research is the biphasic, or bell-shaped, dose-response relationship often termed the Arndt-Schulz curve [91-93]. Below a threshold dose, insufficient stimulus produces no measurable effect. Within an optimal window, maximal beneficial responses occur. Above this window, inhibitory effects emerge, and at sufficiently high doses, damage may result.

This biphasic pattern has been demonstrated for multiple endpoints at cellular, tissue, and organism levels [91-93]. In vitro, ATP production and mitochondrial membrane potential show clear biphasic responses to increasing fluence. In animal wound-healing models, intermediate fluences (approximately 2-10 J/cm²) accelerate healing, while higher fluences (greater than 50 J/cm²) may impede it. Similar patterns appear in anti-inflammatory effects, neural regeneration, and other PBM applications.

**Figure 6:**
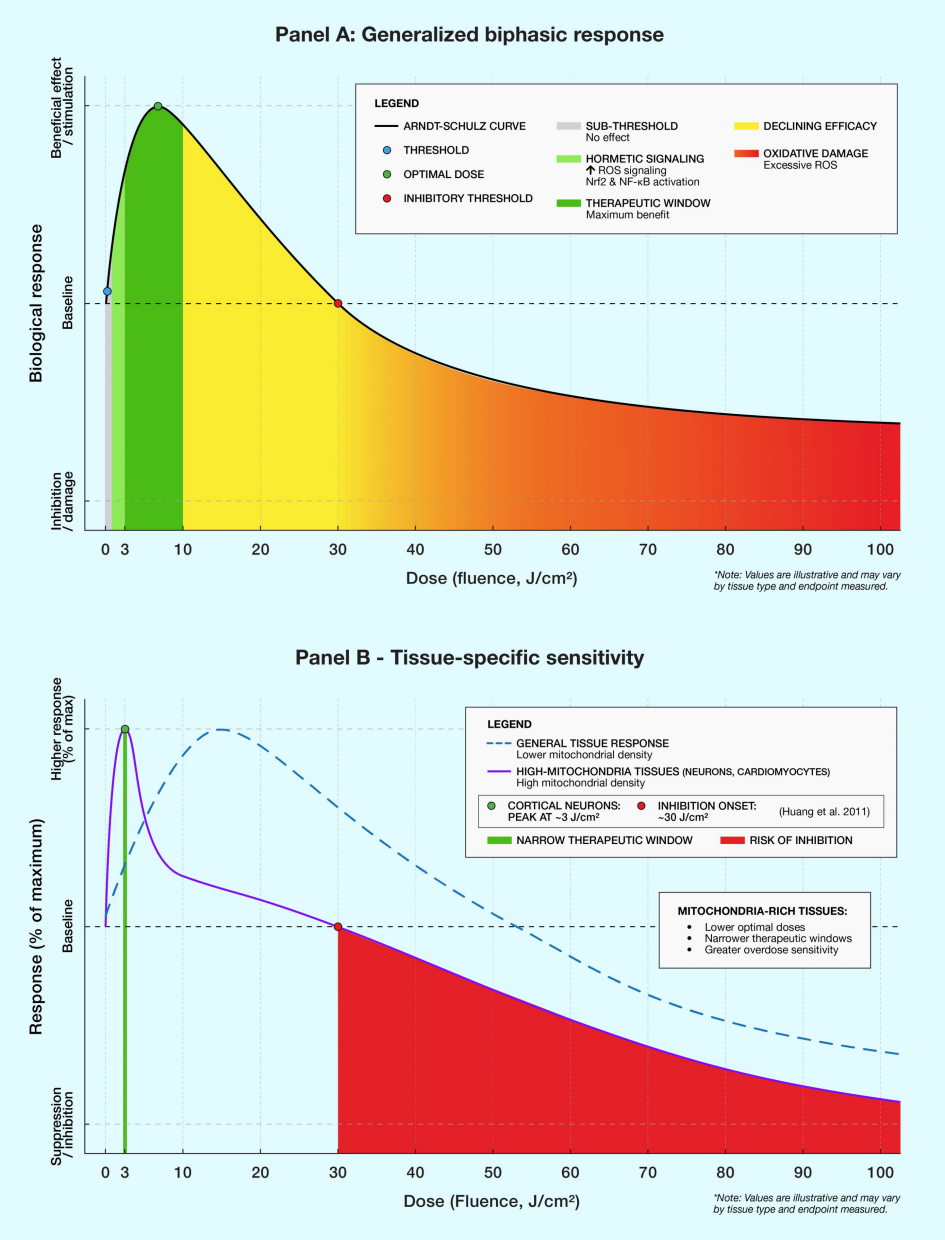
Biphasic dose-response relationship in PBM and tissue-specific sensitivity (A) The Arndt-Schulz curve: Biological responses to PBM follow a bell-shaped rather than linear pattern. Below threshold doses, no measurable effect occurs. Within the optimal therapeutic window, maximal beneficial responses are achieved through ROS-mediated signaling that activates protective transcription factors (Nrf2, NF-κB). Above this window, excessive ROS production overwhelms antioxidant defenses, producing inhibitory effects and potential tissue damage. The therapeutic window (green zone) represents the target range for clinical applications. (B) Tissue-specific sensitivity varies with mitochondrial density. High-mitochondria-density tissues such as neurons exhibit narrower therapeutic windows compared to tissues with lower mitochondrial content. For cortical neurons, peak beneficial effects on ATP production and mitochondrial membrane potential occur at approximately 3 J/cm², with inhibitory effects emerging around 30 J/cm², a 10-fold range. Tissues with lower mitochondrial density may tolerate higher doses with broader therapeutic windows. This variability necessitates tissue-specific protocol optimization and explains why "more is not better" in PBM applications. The narrower margin in mitochondria-rich tissues has important implications for transcranial protocols. PBM: photobiomodulation; ROS: reactive oxygen species; Nrf2: nuclear factor erythroid 2-related factor 2; ATP: adenosine triphosphate Image created by the Mercola graphic design team, located in Manila, Philippines, using Adobe Illustrator and Adobe Photoshop (San Jose, California, United States)

The mechanisms underlying the biphasic dose-response likely involve the Janus nature of ROS [91,92]. At low concentrations, ROS serve as beneficial signaling molecules, activating protective transcription factors including NF-κB and Nrf2. At higher concentrations, ROS overwhelm antioxidant defenses and cause oxidative damage. PBM increases mitochondrial ROS production as part of its signaling mechanism; the transition from beneficial signaling to harmful excess may explain the biphasic response. Figure 6 illustrates the biphasic dose-response relationship characteristic of PBM, emphasizing the narrow therapeutic window in mitochondria-dense tissues such as the brain.

Tissue-specific sensitivity

Optimal dose parameters appear to vary across tissues, with high-mitochondria-density tissues potentially more sensitive to both beneficial and adverse effects [92]. For cortical neurons specifically, Huang and colleagues demonstrated peak ATP production and mitochondrial membrane potential at approximately 3 J/cm² using an 810 nm laser, with inhibitory effects emerging at 30 J/cm², where mitochondrial membrane potential actually fell below baseline [92]. This narrower window compared to some other cell types may reflect neurons' extreme mitochondrial density and limited antioxidant reserves.

The practical implication is that "more is not better" in PBM and optimal dosing may be tissue-specific [91-93]. Transcranial applications must account for light attenuation through the scalp, skull, and meninges while avoiding excessive surface irradiance. The complexity of rationally selecting among wavelength, fluence, power density, and treatment duration may partly explain the inconsistent results across PBM studies. It may indeed be easier to overdose high-mitochondria-density tissues, a consideration that should inform protocol development.

Cumulative effects and treatment intervals

Effects from individual PBM treatments may persist and accumulate. Some studies report effects lasting up to 54 hours post-treatment, suggesting that daily treatments may produce cumulative rather than independent effects [94]. If so, adequate recovery time between sessions may be necessary to avoid exceeding the beneficial dose window through cumulative exposure. This consideration has received limited systematic investigation; studies examining optimal inter-treatment intervals would substantially inform clinical protocol development.

Critical evaluation of biphasic dose-response evidence

The biphasic dose-response relationship is one of the best-established findings in PBM research, having been documented across multiple endpoints and experimental systems [91-93]. This provides important guidance for protocol development. However, the precise parameters defining the therapeutic window vary across studies, tissues, and endpoints, complicating translation to clinical practice. The optimal parameters for transcranial PBM targeting cognitive outcomes in humans have not been definitively established through systematic dose-finding studies.

Potential risks and adverse effects

While PBM is generally considered safe with minimal reported adverse effects, a comprehensive evaluation of the proposed NIR-melatonin-glutathione framework should acknowledge potential risks that warrant investigation.

Theoretical concerns

The same mitochondrial stimulation that could benefit healthy or degenerating neurons might also theoretically benefit tumor cells. Malignant cells often exhibit altered mitochondrial metabolism and high energy demands. Whether PBM could promote tumor growth or resistance to therapy remains inadequately studied. Until evidence demonstrates safety, caution is warranted in patients with known or suspected brain tumors.

Chronic stimulation of any biological pathway raises questions about adaptive responses. Could repeated NIR exposure lead to the downregulation of endogenous protective mechanisms, creating dependency or tolerance? Studies examining the long-term effects of sustained PBM treatment would address this concern.

Individuals with mitochondrial diseases characterized by existing ETC dysfunction might respond differently to PBM than healthy individuals. Whether stimulation of already-compromised mitochondria could be beneficial or harmful requires specific investigation in these populations.

Clinical safety data

The available clinical safety data, while limited, are generally reassuring. Transcranial PBM studies have reported minimal adverse effects, typically limited to mild headache in some participants. The Neurothera Effectiveness and Safety Trial 3 (NEST-3) of transcranial laser therapy for acute stroke, despite failing to demonstrate efficacy, did not identify significant safety concerns [97]. However, most studies have been short-term, and long-term safety data are lacking. The biphasic dose-response underscores that excessive treatment could be counterproductive or harmful. Establishing safe upper limits for fluence, treatment frequency, and cumulative exposure should be a priority for clinical development.

Alzheimer's disease: a primary application

Alzheimer's disease provides a compelling context for evaluating the proposed NIR-melatonin-glutathione framework, as the disease involves documented deficiencies in multiple components of the proposed protective cascade and preliminary clinical evidence supports PBM efficacy.

The mitochondrial cascade hypothesis of Alzheimer's disease

While amyloid plaques and tau tangles have traditionally been considered primary drivers of Alzheimer's disease pathology, mounting evidence supports mitochondrial dysfunction as an early and potentially causative event [21,22]. CCO deficiency is the most consistently identified ETC abnormality in Alzheimer's disease brain tissue, present even in regions without plaque or tangle pathology [22]. Reduced CCO activity decreases ATP availability for synaptic function while simultaneously increasing electron leakage and superoxide production. This creates a potential vicious cycle: mitochondrial dysfunction increases oxidative stress, which damages mitochondrial components, which further impairs function.

The mitochondrial cascade hypothesis proposes that amyloid accumulation may represent a downstream consequence rather than the primary cause of neurodegeneration. Mitochondrial dysfunction reduces clearance capacity for Aβ while simultaneously increasing its production through effects on APP processing. If this model is correct, interventions targeting mitochondrial function rather than amyloid directly may prove more effective than the amyloid-focused therapies that have largely failed in clinical trials.

Melatonin deficiency in Alzheimer's disease

Cerebrospinal fluid melatonin concentrations in Alzheimer's patients are profoundly reduced, approximately 20% of age-matched control levels [12,13]. Liu et al. [12] and Zhou et al. [13] demonstrated that this reduction appears at the earliest neuropathological stages (Braak I-II). Pineal calcification, which reduces melatonin production, is also significantly more prevalent in Alzheimer's patients compared to controls.

Whether melatonin deficiency contributes to disease pathogenesis or represents a consequence of neurodegeneration remains uncertain. However, the early timing of melatonin decline, preceding clinical symptoms, and melatonin's documented protective effects against amyloid toxicity and tau hyperphosphorylation suggest a potential causal role. These findings support but do not prove a causal relationship; prospective studies examining whether melatonin decline predicts Alzheimer's development would be informative.

Clinical evidence for PBM in cognitive decline

Preliminary clinical trials support PBM efficacy in Alzheimer's disease and related dementias. Table 3 summarizes published and ongoing clinical studies evaluating PBM in Alzheimer's disease and cognitive decline.

**Table 3:** Clinical studies evaluating PBM for AD and cognitive decline Studies are organized by design type. While results in dementia populations are consistently positive, sample sizes remain small and methodological rigor varies. MMSE: Mini-Mental State Examination; RCT: randomized controlled trial; PBM: photobiomodulation; BDNF: brain-derived neurotrophic factor; TRAP-AD: Transcranial Photobiomodulation for Alzheimer's Disease; MCI: mild cognitive impairment; AD: Alzheimer's disease; NEST-3: Neurothera Effectiveness and Safety Trial 3

Study	Design	Sample	Treatment parameters	Key outcomes	Reference
Saltmarche et al. (2017)	Case series	n=5 mild-moderate dementia	Transcranial + intranasal, 12 weeks	Significant MMSE improvement; caregiver-reported functional gains	[45]
Chao (2019)	Pilot RCT	n=8 dementia	Home-based transcranial PBM	Improved cognition, behavior, cerebral perfusion, default mode network connectivity	[46]
de Oliveira et al. (2024)	RCT, double-blind, placebo-controlled	Adults >50 years	Transcranial PBM	Cognitive improvement; increased serum BDNF	[47]
De Taboada et al. (2011)	Animal study	APP transgenic mice	Transcranial laser	Reduced amyloid plaque burden; improved memory	[98]
TRAP-AD (ongoing)	RCT	MCI and early AD	Transcranial PBM, dose-finding	Neuroimaging endpoints (cerebral blood flow, connectivity)	[99]
NEST-3 (2014)	Phase III RCT	Acute stroke (n=1000)	Single transcranial laser treatment	No efficacy demonstrated; no significant safety concerns	[97]

Saltmarche and colleagues reported significant cognitive improvement in five dementia patients treated with transcranial plus intranasal PBM for 12 weeks [45]. Chao conducted a randomized controlled trial in eight dementia patients, demonstrating improvements in cognition, behavior, cerebral perfusion, and default mode network connectivity in the PBM group compared to usual care [46]. More recently, de Oliveira and colleagues reported cognitive improvements and increased serum brain-derived neurotrophic factor (BDNF) in a randomized, double-blind, placebo-controlled trial of transcranial PBM in adults over 50 [47].

However, the clinical evidence must be interpreted cautiously. The NEST-3, a phase III clinical trial of transcranial laser therapy for acute stroke, failed to demonstrate efficacy, raising questions about the translatability of preclinical PBM findings to human neurological disease [97]. Several factors may explain this discrepancy between preclinical promise and clinical failure: acute stroke involves ischemic tissue where mitochondrial function is already severely compromised, potentially limiting CCO-mediated responses; the single-treatment paradigm employed in NEST-3 differs fundamentally from the cumulative effects hypothesized for chronic neurodegeneration prevention; and optimal parameters including wavelength, fluence, and timing may differ substantially between acute rescue interventions and chronic protective applications. Nevertheless, this null result in a large, well-designed phase III trial underscores that preclinical PBM findings do not automatically translate to clinical efficacy and highlights the notable importance of rigorous human trials before drawing conclusions about therapeutic utility.

Animal studies have demonstrated that PBM reduces amyloid plaque burden and improves memory in transgenic AD models [98]. Larger and more rigorous trials are ongoing, including the TRAP-AD study at New York University (NYU) evaluating the dose-dependent effects of transcranial PBM in mild cognitive impairment and early Alzheimer's disease using neuroimaging endpoints [99].

These findings, while promising, must be interpreted cautiously given the small sample sizes and methodological limitations in early studies. The clinical evidence remains preliminary, and null findings or adverse effects may exist in unpublished studies. Nevertheless, the convergence of mechanistic rationale with early clinical signals provides a compelling case for continued investigation.

Translational considerations and alternative mechanisms

The clinical evidence summarized above demonstrates that transcranial PBM can produce measurable cognitive and neurobiological effects in preliminary trials. However, these findings do not establish melatonin mediation. None of the cited PBM trials measured melatonin as a primary or secondary endpoint, and the observed benefits could be explained by mechanisms independent of the proposed melatonin cascade.

Alternative mechanisms with independent experimental support include the following: increased cerebral blood flow and oxygenation, as demonstrated by NIR spectroscopy studies showing hemodynamic changes following transcranial NIR [52,53]; modulation of neuroinflammation through effects on microglial polarization and cytokine profiles [51]; direct NO signaling following photodissociation from CCO, with downstream effects on vascular tone and synaptic plasticity independent of melatonin [33,34]; upregulation of BDNF, as documented in the de Oliveira et al. trial [47]; and enhanced cerebral lymphatic and glymphatic drainage [100].

These alternative mechanisms and the proposed melatonin-mediated pathway are not mutually exclusive. NIR absorption by CCO simultaneously releases NO, increases ATP production, generates signaling-level ROS, and may (as hypothesized here) stimulate local melatonin synthesis. The relative contribution of each mechanism to observed clinical benefits is unknown and likely varies with treatment parameters, target tissue, and patient characteristics. Future trials designed to distinguish between these mechanisms, for example, by measuring melatonin and glutathione biomarkers alongside cognitive endpoints, will be essential for determining whether the proposed cascade contributes to PBM efficacy.

The glymphatic connection

The glymphatic system provides an additional mechanism by which NIR and melatonin may protect against Alzheimer's disease pathology. This perivascular clearance pathway facilitates the cerebrospinal fluid-mediated removal of extracellular solutes including Aβ and tau [29]. Glymphatic function is dramatically enhanced during sleep, particularly slow-wave sleep, and suppressed during wakefulness [101,102].

Impaired glymphatic function has been linked to accelerated amyloid accumulation, and sleep disruption, common in Alzheimer's disease, correlates with increased cerebrospinal fluid Aβ levels [103]. Huang et al. [103] demonstrated that glymphatic system dysfunction predicts amyloid deposition, neurodegeneration, and clinical progression in Alzheimer's disease. Studies in mice suggest that glymphatic clearance of Aβ approximately doubles during sleep compared with waking. Conversely, interventions enhancing glymphatic clearance might reduce amyloid burden. Murdock et al. [104] showed that multisensory gamma stimulation promotes glymphatic clearance of Aβ. Salehpour et al. [100] reported that PBM has been shown to enhance cerebral blood flow, cerebrospinal fluid dynamics, and meningeal lymphatic function. If pre-sleep PBM can prime glymphatic function for enhanced overnight clearance, it would provide a mechanism for neurodegeneration prevention distinct from but complementary to daytime mitochondrial protection. However, this proposed link between PBM and glymphatic enhancement rests on limited evidence and requires direct experimental validation.

Critical evaluation of Alzheimer's disease application evidence

The evidence supporting mitochondrial dysfunction in Alzheimer's disease is robust [21,22], as is the documentation of melatonin deficiency [12,13]. The preliminary clinical trials of PBM in dementia show consistently positive results [45-47], but sample sizes are small (typically <20 participants), and methodological rigor varies. The failure of NEST-3 in acute stroke [97] provides an important counterpoint, though the clinical contexts differ substantially. So, the glymphatic connection is intriguing but speculative. Overall, Alzheimer's disease represents a rational target for the proposed intervention, but definitive efficacy data from large, well-controlled trials are lacking.

Knowledge gaps and future directions

Despite substantial evidence supporting individual components of the proposed framework, several significant questions remain unanswered. Addressing these gaps will be essential for translating mechanistic understanding into clinical applications.

Mechanistic Priorities

The most significant mechanistic gap is the lack of direct demonstration of NIR-induced mitochondrial melatonin synthesis in human tissue. While CCO photobiology and mitochondrial melatonin synthesis capacity have been independently established, studies explicitly tracing the bicarbonate/calcium→sAC→cAMP→AANAT cascade in response to NIR stimulation would substantially strengthen the integrated hypothesis. Such studies would require sophisticated metabolic labeling and subcellular fractionation techniques applied to intact tissue following controlled NIR exposure.

The automitocrine hypothesis, which posits that mitochondria possess melatonin receptors and respond to locally produced melatonin, requires independent confirmation. The initial report of mitochondrial MT1 localization has not been extensively replicated, and alternative explanations have not been definitively excluded. High-resolution imaging studies and receptor knockout experiments would clarify this important mechanism.

The relationship between NIR parameters (wavelength, fluence, timing) and mitochondrial melatonin output requires systematic characterization. Current PBM protocols have been developed empirically based on functional endpoints; understanding the melatonin synthesis response would permit more rational protocol optimization.

Clinical Priorities

Randomized controlled trials with adequate sample sizes and rigorous blinding are urgently needed to establish PBM efficacy in neurodegenerative disease. Multi-center trials with standardized protocols and validated cognitive endpoints would provide definitive efficacy data.

Long-term follow-up studies are essential for evaluating neuroprotection. Short-term cognitive improvements, while encouraging, do not necessarily indicate disease-modifying effects. Studies following participants for years, ideally with biomarker and imaging endpoints, would determine whether PBM slows disease progression or merely provides symptomatic benefit.

Dose-optimization studies are needed across wavelengths, power densities, treatment schedules, and delivery modalities. The biphasic dose-response relationship makes the selection of optimal parameters vital for efficacy. Head-to-head comparisons of different protocols would identify the most effective approaches.

Equally important, clinical trials of PBM for neuroprotection should address the substrate requirements for the proposed glutathione amplification cascade. The framework assumes that melatonin-induced enzyme activation will increase glutathione production, but this can only occur if glycine and cysteine are available in sufficient quantities. Given robust evidence that older adults commonly exhibit deficiencies in these precursors and that these deficiencies impair glutathione synthesis even when enzymatic capacity is preserved [28,30], future trials should consider incorporating glutathione precursor supplementation. Based on clinical evidence, appropriate daily doses would be approximately 10 g of glycine and 1.2 g of NAC (600 mg twice daily) to ensure substrate adequacy [28,29]. This approach would ensure that any NIR-mediated increase in mitochondrial melatonin synthesis can translate into the predicted downstream amplification of glutathione-based antioxidant defense. Trials comparing PBM alone versus PBM combined with glycine and NAC supplementation would directly test whether substrate availability limits therapeutic response and could identify a synergistic intervention strategy superior to either approach alone.

Comparative Questions

Time-of-day effects on PBM efficacy also require systematic investigation in humans. If circadian variation in CCO responsiveness translates from animal models, treatment timing could be an important but currently overlooked parameter. Individual variation factors, including age, genetics, baseline mitochondrial health, and existing disease, likely influence PBM response. Identifying predictors of response would permit personalized treatment approaches and help explain heterogeneous results in clinical trials.

Limitations

As a narrative hypothesis paper, this work provides an interpretive synthesis rather than exhaustive systematic coverage. Study selection reflects the author's judgment and may not capture all relevant literature. The emphasis on English-language publications may have excluded important non-English studies. The integrative approach, while permitting novel synthesis across traditionally separate fields, may underemphasize within-field controversies or methodological debates.

The hypothesis presented, while mechanistically plausible and consistent with available evidence, represents a synthesis that has not been experimentally validated as a unified system. Individual components have varying levels of support: CCO photobiology and melatonin antioxidant properties are well-established, mitochondrial melatonin synthesis is supported by strong recent evidence, but the integrated NIR-responsive cascade remains hypothetical. Readers should recognize the distinction between established findings and proposed mechanisms.

Limitations of the available evidence

The evidence base informing this hypothesis has several significant limitations. Many foundational PBM studies used isolated mitochondria or cell culture systems, and translation to intact organisms introduces additional complexity. In vivo confirmation of CCO-mediated mechanisms in mammalian brain tissue, while supported by indirect evidence, requires additional direct measurement.

Clinical evidence for PBM in neurodegeneration derives predominantly from small, single-center studies with limited follow-up. Publication bias favoring positive results may inflate apparent efficacy. Negative or null findings from unpublished trials cannot be excluded. The heterogeneity of protocols across studies, varying in wavelength, power, treatment schedule, and delivery method, complicates synthesis and comparison. The failure of the NEST-3 in acute stroke provides an important cautionary note about extrapolating from preclinical and pilot clinical findings.

The relationship between melatonin and Alzheimer's disease remains correlational. While reduced cerebrospinal fluid melatonin is consistently observed, causality has not been established. Melatonin decline could represent a consequence of neurodegeneration rather than a contributing factor, in which case melatonin restoration might not provide disease-modifying benefit.

Long-term outcomes from PBM have not been established. Predictions about long-term neuroprotection, while consistent with mechanistic rationale, remain hypothetical extrapolations.

These limitations suggest that conclusions regarding specific mechanisms should be considered preliminary pending higher-quality evidence. The strongest conclusions concern the established antioxidant properties of melatonin and the documented effects of PBM on mitochondrial function. The most speculative elements involve the integrated cascade's operation as a unified NIR-responsive system and predictions about long-term neuroprotection. Readers should calibrate confidence accordingly.

Future directions

If validated, this framework suggests that PBM represents the restoration of an evolutionarily ancient, light-dependent protective system rather than the introduction of a novel therapy. However, this therapeutic framing is contingent on the experimental validation of the central hypothesis, that NIR radiation triggers mitochondrial melatonin synthesis, which remains the least-established link in the proposed framework. The practical implications include non-invasive, targeted neuroprotection with minimal adverse effect potential. The mechanistic framework suggests that treatment optimization should consider timing (morning exposure aligned with CCO responsiveness peaks), dosing (within the optimal therapeutic window avoiding biphasic inhibition), frequency (permitting adequate inter-treatment recovery), and substrate status (concurrent glycine/NAC supplementation to ensure glutathione synthesis capacity).

Although this paper focuses on neuroprotection, the proposed NIR-melatonin-glutathione cascade should theoretically operate in any mitochondria-rich tissue, suggesting potential applications in cardiac protection, renal protection, and other contexts that warrant future investigation.

The evolutionary context deserves emphasis. Modern indoor living has eliminated the NIR exposure humans experienced throughout evolutionary history. If NIR serves as a physiological stimulus for endogenous protective mechanisms, contemporary humans may be experiencing a novel form of environmental deficiency with consequences for brain health. PBM would then represent not the introduction of a novel therapy but the restoration of an ancient, sun-dependent protective system that modern lifestyles have inadvertently disrupted.

Within this context, the proposed framework identifies several priorities for future research:

Mechanistic Validation

The most critical gap is the direct demonstration of NIR-induced mitochondrial melatonin synthesis in human tissue. Studies using isotope labeling, real-time imaging, or genetic manipulation to trace the complete NIR→CCO→sAC→cAMP→AANAT→melatonin pathway would substantially strengthen or refute the core hypothesis.

Parameter Optimization

Systematic characterization of dose-response relationships across wavelengths (630-1100 nm), fluences (1-50 J/cm²), power densities, treatment timing, and inter-session intervals is needed. The biphasic response and tissue-specific sensitivity require careful attention to avoid subtherapeutic or excessive dosing.

Clinical Translation

Large, multi-center randomized controlled trials with standardized protocols, validated cognitive and biomarker endpoints, and long-term follow-up (≥2 years) are essential to establish disease-modifying effects rather than symptomatic benefits.

Substrate Interactions

Trials comparing PBM alone versus PBM combined with glycine/NAC supplementation would test whether substrate availability limits therapeutic response and could identify synergistic intervention strategies.

Individual Variation

Identification of predictors of PBM response (age, genetics, baseline mitochondrial function, substrate status, disease stage) would enable personalized treatment approaches and explain heterogeneous results across studies.

Concluding remarks

The next decade of research will determine whether PBM takes its place among evidence-based strategies for healthy brain aging or remains an intriguing but unproven hypothesis. The mechanistic framework proposed here, while requiring experimental validation of its integrated components, provides a foundation for designing the rigorous studies needed to resolve this question.

Ultimately, the NIR-mitochondrial melatonin-glutathione cascade represents both a significant scientific challenge and an important opportunity. Understanding the mechanisms by which light interacts with brain biology could open new avenues for preventing the cognitive decline that represents one of the most significant healthcare challenges of aging populations worldwide.

## Conclusions

This hypothesis paper proposes that NIR radiation activates a mitochondrial protection cascade through CCO photostimulation, culminating in local melatonin synthesis and comprehensive antioxidant defense via glutathione amplification and SIRT3-mediated SOD2 activation. The evidence synthesis reveals three tiers of support.

For well-established foundations (★★★★), CCO serves as a primary NIR chromophore, supported by action spectrum matching, photodissociation studies, and functional demonstrations in multiple experimental systems. Melatonin exerts potent mitochondrial antioxidant effects through both direct radical scavenging and the upregulation of glutathione-related enzymes. Mitochondria possess complete melatonin-synthesizing machinery (AANAT, ASMT) independent of pineal regulation, as demonstrated by isotope-labeling experiments. Age-related glutathione deficiency can be corrected by glycine and NAC supplementation. FOXO3 genetic variants are robustly associated with human longevity across multiple populations.

For supported but preliminary findings (★★★☆), transcranial NIR reaches brain mitochondria and increases CCO activity, as demonstrated by NIR spectroscopy in humans. Preliminary clinical trials show cognitive benefits in dementia patients treated with PBM. Melatonin activates SIRT3-FOXO3a signaling across multiple tissue models including cardiac, intestinal, and pulmonary systems. Circadian variation in PBM efficacy has been demonstrated in retinal tissue.

For hypothesized but untested connections (★★☆☆ to ★☆☆☆), NIR-induced bicarbonate/sAC/cAMP signaling triggering mitochondrial melatonin synthesis represents a mechanistically plausible but experimentally unvalidated pathway. Substrate availability (glycine, cysteine) as a determinant of variable PBM response has not been directly tested. The automitocrine hypothesis regarding mitochondrial melatonin receptors requires independent confirmation. The complete integrated cascade operating as a unified NIR-responsive system in human brain tissue has not been demonstrated. The hypothesized link between NIR exposure and mitochondrial melatonin synthesis represents the least validated connection in the proposed cascade and constitutes the single most important experimental gap to be addressed by future research.

To advance this framework from a plausible hypothesis to a validated mechanism, five priority research questions require experimental investigation: First, does transcranial NIR exposure increase mitochondrial melatonin concentration in human cortical tissue, as measurable by microdialysis in neurosurgical patients or analysis of post-surgical tissue samples? Second, does NIR exposure increase AANAT enzymatic activity in human-derived neuronal cell cultures, and is this effect dependent on mitochondrial soluble adenylyl cyclase activity? Third, does concurrent supplementation with glycine and NAC potentiate PBM effects on peripheral redox biomarkers (plasma GSH-to-GSSG ratio, 4-HNE, 8-OHdG) in a randomized controlled trial of older adults? Fourth, do individuals with higher baseline plasma glycine and cysteine levels show greater cognitive response to standardized transcranial PBM protocols than substrate-depleted individuals? Fifth, does morning transcranial PBM produce greater effects on cerebrospinal fluid melatonin or cortical oxidized CCO concentration (measured by broadband NIR spectroscopy) than afternoon PBM in the same subjects using a crossover design?

Affirmative answers to these questions would substantially strengthen the proposed framework; negative findings would identify which components require revision or abandonment.
